# p32/C1QBP regulates OMA1-dependent proteolytic processing of OPA1 to maintain mitochondrial connectivity related to mitochondrial dysfunction and apoptosis

**DOI:** 10.1038/s41598-020-67457-w

**Published:** 2020-06-30

**Authors:** Solhee Noh, Sophors Phorl, Rema Naskar, Kakada Oeum, Yuri Seo, Eunjung Kim, Hee-Seok Kweon, Joo-Yong Lee

**Affiliations:** 1 Graduate School of Analytical Science and Technology (GRAST), Chungnam National University, 99 Daehak-ro(St), Yusoeng, Taejon, 305-764 Republic of Korea; 2 Center for Research Equipment, Korea Basic Science Institute, Cheongju, 28119 Korea; 3 Korea Basic Science Institute, Taejon, 34133 Republic of Korea

**Keywords:** Mitochondria, Energy metabolism, Apoptosis

## Abstract

Mitochondria are dynamic organelles that undergo fusion and fission in response to various physiological and stress stimuli, which play key roles in diverse mitochondrial functions such as energy metabolism, intracellular signaling, and apoptosis. OPA1, a mitochondrial dynamin-like GTPase, is responsible for the inner membrane fusion of mitochondria, and the function of OPA1 is regulated by proteolytic cleavage in response to various metabolic stresses. Growing evidences highlighted the importance of mitochondrial adaptation in response to metabolic stimuli. Here, we demonstrated the role of p32/C1QBP in mitochondrial morphology by regulating OMA1-dependent proteolytic processing of OPA1. Genetic ablation of p32/C1QBP activates OMA1, cleaves OPA1, and leads mitochondrial fragmentation and swelling. The loss of p32/C1QBP decreased mitochondrial respiration and lipid utilization, sensitized cells to mitochondrial stress, and triggered a metabolic shift from oxidative phosphorylation to glycolysis, which were correlated with apoptosis in cancer cells and the inhibition of 3D-spheroid formation. These results suggest a unique regulation of cell physiology by mitochondria and provide a basis for a new therapeutic strategy for cancer.

## Introduction

Mitochondrial morphology varies widely among many cell types and tissues, changing rapidly in response to metabolic alterations, such as nutrient status^1–3^. Mitochondria do not operate as a single separated organelle. Rather, they function as a group whose activity is coordinated by mitochondrial dynamics^4^. The term "mitochondrial dynamics" includes behaviors of mitochondria, such as their membrane fusion and fission, their transport along the cytoskeleton, their selective degradation by the mitophagy pathway, and their interactions with other organelles, including the endoplasmic reticulum^4^. Among them, a continuous cycle of mitochondrial membrane fusion and fission serves to mix the contents of the mitochondrial population, promote homogeneity of organelles, control the morphology of mitochondria, and maintain high functionality^4^. An unobstructed mitochondrial fusion leads to a hyperfused mitochondrial network to counteract metabolic insults, preserve cellular integrity, and protect against mitophagy and apoptosis^1, 5^. In this view, mitochondrial fusion corresponds with an increased mitochondrial ATP production, while its inhibition corresponds with increased mitochondrial ROS and impaired OXPHOS^1, 3, 6^.

The complement C1q binding protein (p32/C1QBP, hereafter referred to as p32) is localized predominantly in the mitochondrial matrix^7, 8^ and acts as a multifunctional chaperone protein required for the formation of mitochondrial ribosome complexes^9^. Considering this, p32 is essential for the maintenance of the respiratory chain complex and OXPHOS in cancer cells^10^ and abundantly expressed in various types of cancers^11–17^. In neuronal cells, p32 was involved in mitochondrial morphology and dynamics, through Parkin^18^. However, it is still unknown if p32 controls the mitochondrial morphology in diverse cell types that did not express Parkin, unlike the neuronal cells. If p32 does control the mitochondrial morphology, it is not clear which mitochondrial fusion protein is responsible and the underlying molecular mechanism.

Because mitochondria have a double membrane (outer- and inner-membrane), mitochondrial fusion consists of two membrane fusion events: outer membrane fusion, followed by inner membrane fusion^4^. In mammals, outer membrane fusion is mediated by two mitofusins, mitofusin 1 (MFN1) and mitofusin 2 (MFN2)^19^. In the absence of MFN1 and MFN2, neither outer nor inner membrane fusion occurs^19, 20^. Inner membrane fusion is mediated by OPA1 mitochondrial dynamin-like GTPase (OPA1)^21^. In the absence of OPA1, outer membrane fusion occurs, but inner membrane fusion does not^20^. Notably, OPA1 also maintains a negative cristae junction curvature in the mitochondrial inner membrane, which controls cytochrome C redistribution and release^22, 23^, and stabilizes the respiratory super-complexes to increase respiratory efficiency^24, 25^. OPA1 has been studied intensely in apoptosis associated with cytochrome C release.

OPA1 functions are controlled mainly by proteolysis via two mitochondrial proteases, the ATP-independent protease OMA1 (OMA1 zinc metallopeptidase), and the ATP-dependent AAA+protease YME1L (YME1 like 1 ATPase). Long membrane-bound active form of OPA1 has two protease cleavage sites, S1 (cleaved by OMA1), and S2 (cleaved by YME1L), to generate short inactive forms of OPA1, resulting in fusion defects of the inner mitochondrial membrane^26, 27^. YME1L is constitutively active, but OMA1 is activated in response to mitochondrial stress, such as depolarization of mitochondrial membrane^28, 29^. OMA1 and YME1L coordinately regulate mitochondrial fusion and cristae structure by differential proteolytic processing of OPA1^30^.

Although multiple roles of p32 in mitochondria have been studied, it is not clear how p32 regulates mitochondrial morphology and the resulting physiological consequences. Here, we show that p32 controls mitochondrial morphology by regulating the OMA1-dependent proteolytic processing of OPA1. The loss of p32 reduces mitochondrial respiration and sensitizes mitochondria to metabolic stress, which induces a metabolic shift from OXPHOS to glycolysis, resulting in glucose addiction related with apoptosis in cancer cells and 3D spheroid formation.

## Results

### p32/C1QBP controls the proteolytic processing of OPA1 to regulate mitochondrial morphology

**Figure 1:**
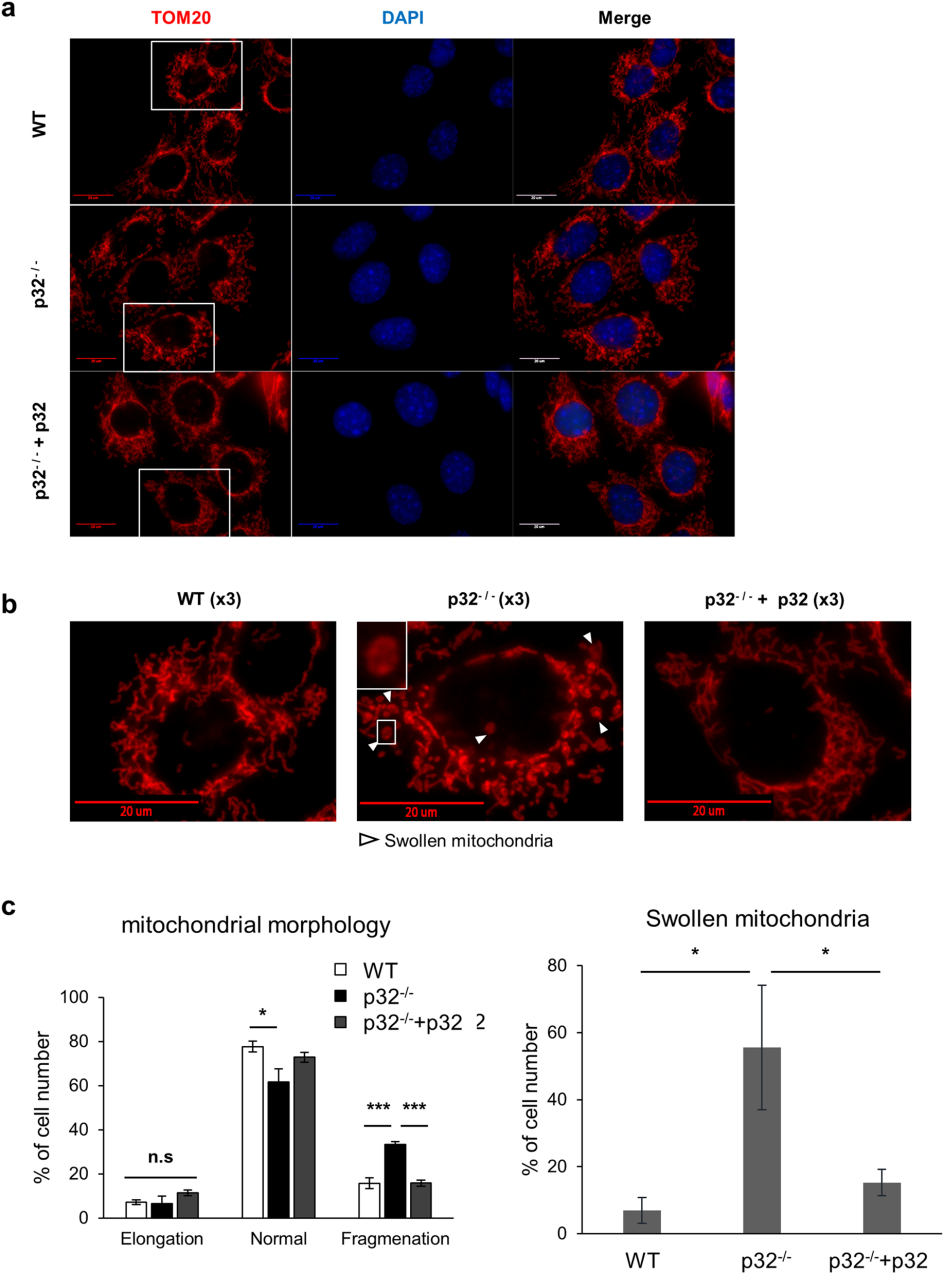
p32/C1QBP prevents mitochondrial fragmentation and swelling. Immunofluorescent staining of wild type, p32^−/−^, and p32^−/−^+p32 MEFs with TOM20 antibody (mitochondria) and DAPI (nuclear). (**a**) Representative images were shown. Scale bar 20 μm. (**b**) Boxed areas in (**a**) were enlarged to show fragmented and swollen mitochondria clearly (arrowhead). Scale bar 20 μm. (**c**) Quantification of results from mitochondrial morphology and swelling. Cells with indicated mitochondrial morphology (left panel) or swollen mitochondria (right panel) were counted and results presented as % of cell number from 3 independent experiments, and at least 100 cells were counted in each experiment. The error bars indicate SD. Student’s t test, *p < 0.05, ***p < 0.001, *n.s*. non-significant.

The role of p32 in the regulation of mitochondrial membrane fusion was explored by monitoring mitochondrial morphology in wild type, p32^−/−^, and p32^−/−^+p32 mouse embryonic fibroblasts (MEFs). First, mitochondrial localization of ectopically expressed p32 was confirmed by mitochondrial fractionation analysis (SI Figure S1a). Figure 1a shows fragmented mitochondria in p32^−/−^ MEFs; restoration of p32 expression in p32^−/−^ MEFs reverses this fragmentation. Interestingly, the number of cells with swollen mitochondria are significantly higher in p32^−/−^ MEFs, and this effect was reversed by p32 expression (Fig. 1b). These conclusions were supported by results from the statistical analysis of data from three independent experiments (Fig. 1c). We also performed a computer-based image analysis to measure mitochondrial length, width and the ratio of width to length^31^. The average mitochondrial length was slightly changed by p32 expression, but it was not statistically significant (SI Fig. S1b). In contrast, mitochondrial width and the ratio of width to length significantly increased in p32^−/−^ MEFs, which is reversed by the reintroduction of p32 (SI Fig. S1c–e). Mitochondrial fragmentation could result from defective mitochondrial fusion or enhanced mitochondrial fission.

**Figure 2:**
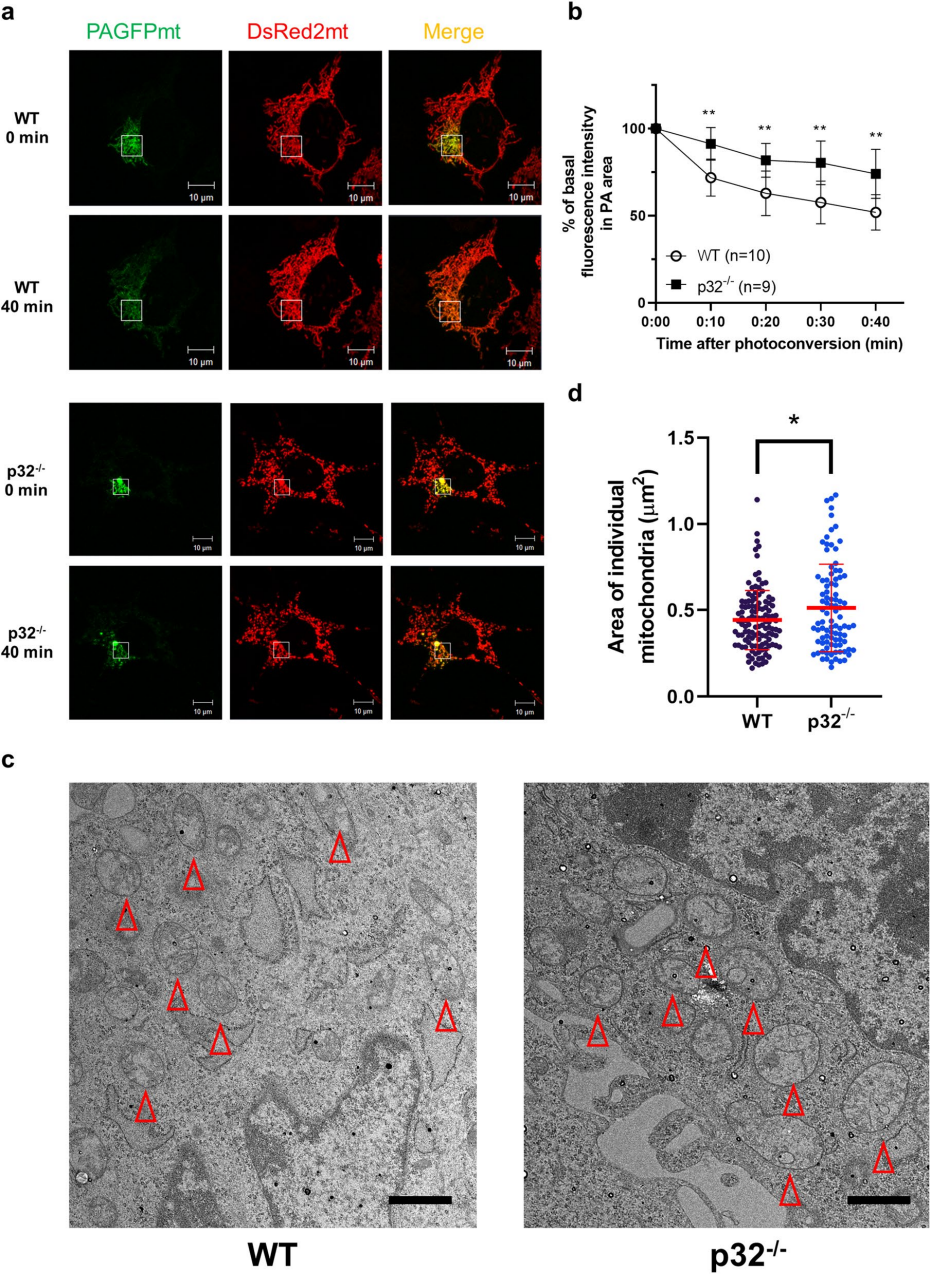
Mitochondrial fusion defect and swelling were observed in p32/C1QBP knockout MEFs. (**a**) Both photoactivatable GFP (PA-GFPmt) and DsRed2mt both targeted to the mitochondrial matrix were expressed in WT and p32^−/−^ MEFs. Mitochondrial fusion was monitored by the time-dependent dilution and redistribution of PA-GFPmt fluorescence. (**b**) Quantification of mitochondrial fusion. Results are represented as % of basal fluorescence intensity in photoactivated area (PA) at indicated time; n = 10 (WT), n = 9 (p32^−/−^). The error bars indicate SD. Student’s t test, **p < 0.01. (**c**) Representative electron microscope images of WT and p32^−/−^ MEFs. Red arrow heads indicates mitochondria. Scale bar is 1 μm. (**d**) Area of individual mitochondrion in electron microscope images were measured by ImageJ and presented as dot plots. The horizontal lines in middle represent the means and error bars indicate ± SD. Student’s t test, *p < 0.05.

To directly assess mitochondrial fusion, we used the PAGFPmt fusion assay^30, 32^. In brief, we ectopically expressed a photoactivable GFP targeted to the mitochondrial matrix (PAGFPmt) in wild type and p32^−/−^ MEFs. Upon photoactivation of a small fraction of mitochondria, we examined fusion of mitochondria by tracking PAGFPmt over time up to 40 min (Fig. 2a, SI Fig. S2). Mitochondrial fusion activity is measured as the reduction of the photoconverted green signal (average intensity) in mitochondria (Fig. 2b). In p32^−/−^ MEFs, PAGFPmt fluorescence intensity was decrease slowly and spread over a smaller area compared with wild type MEFs indicating that mitochondrial fusion is reduced by the genetic abolition of p32 (Fig. 2a,b). We also examined the ultrastructure of mitochondria in p32^−/−^ MEFs by transmission electron microscopy (Fig. 2c). In comparison with wild type, the size of mitochondria was increased in p32^−/−^ MEFs indicating a swollen mitochondrial morphology (Fig. 2c,d).

**Figure 3:**
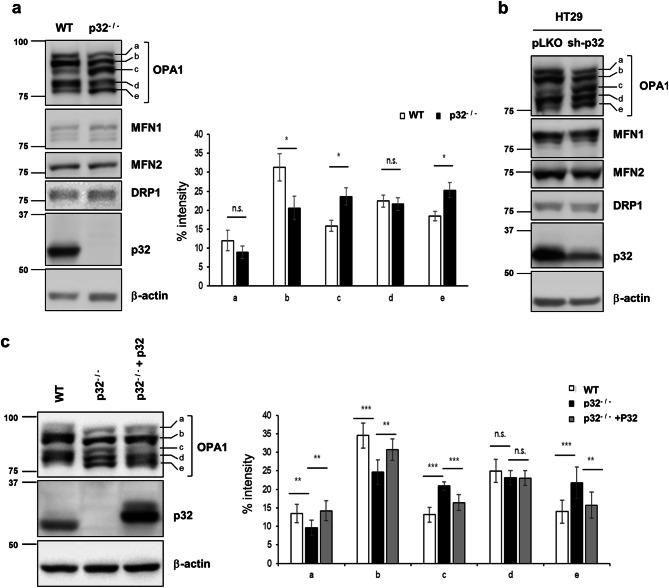
p32/C1QBP inhibits proteolytic cleavages of OPA1. (**a**) Representative western blot analysis of the wild type and p32^−/−^ MEFs for OPA1, MFN1, MFN2, DRP1, p32, and β-actin proteins (right panel). The relative intensity of OPA1 a-, b-, c-, d- and e-form in wild type and p32^−/−^ MEFs were quantified from 3 independent experiments and presented as % intensity (left panel). The error bars indicate SD. Student’s t test, *p < 0.05, *n.s.* non-significant. (**b**) HT29 cells infected with pLKO or sh-p32 lentivirus were selected with puromycin for 1 week to generate stable knockdown cells. The cells were subjected to Western blot analysis for OPA1, MFN1, MFN2, DRP1, p32, and β-actin. (**c**) Wild type and p32^−/−^ MEFs were transfected with pcDNA or a p32 expression vector as indicated and subjected to western analysis for OPA1, p32, and β-actin. The relative intensity of OPA1 a-, b-, c-, d- and e-form were quantified from 4 independent experiments and presented as % intensity (left panel). The error bars indicate SD. Student’s t test, **p < 0.05, ***p < 0.001, *n.s*. non-significant.

To investigate the molecular mechanisms underlying the mitochondrial fragmentation in p32^−/−^ MEFs, we initially measured the levels of Parkin, mitochondrial fusion (OPA1, MFN1, and MFN2) and fission proteins (DRP1) in wild type and p32^−/−^ MEFs by Western blotting. Parkin did not express in wild type and p32^−/−^ MEFs indicating that p32 did not regulate mitochondrial morphology through parkin (SI Fig. S1f). Figure 3a shows in the p32^−/−^ MEFs, only OPA1 immunoblot showed a different cleavage pattern with more cleaved forms of OPA1 (c- and e-form) and less uncleaved form of OPA1 (a- and b-form), suggesting that OPA1 undergoes proteolytic cleavage in p32^−/−^ MEFs. This result was also confirmed by p32 knockdown in HT29 cell line (Fig. 3b). To confirm the role of p32 in the proteolytic cleavage of OPA1, p32 was reintroduced into p32^−/−^ MEFs. As shown in Fig. 3c, the reintroduced p32 successfully reversed the excessive proteolytic cleavage of OPA1 in p32^−/−^ MEFs. Together, the loss of p32 causes the excessive proteolytic processing (c-, e-form) of OPA1 related with changes of mitochondrial morphology.

### p32/C1QBP controls the OMA1-dependent proteolytic cleavage of OPA1

**Figure 4:**
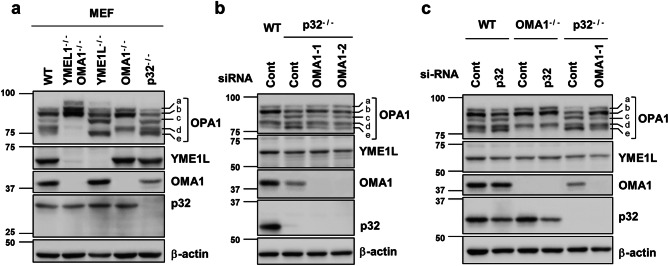
OMA1 causes proteolytic cleavage of OPA1 induced by p32/C1QBP deficiency. (**a**) Wild type, OMA1^−/−^, YME1L^−/−^, OMA1^−/−^YME1L^−/−^, and p32^−/−^ MEFs were analyzed by immunoblotting for the indicated proteins. (**b**) Wild type and p32^−/−^ MEFs were transfected with control or two independent siRNA for OMA1 (OMA1-1 and OMA1-2) and subjected to western blotting for the indicated proteins. (**c**) Wild type, OMA1^−/−^, and p32^−/−^ MEFs were transfected with indicated siRNA. Cell lysates were subjected to Western blotting for indicated proteins.

OMA1 and YMEL1 are the main proteolytic enzymes involved in the cleavage of OPA1 in mitochondria^26, 27^. To identify the mitochondrial protease responsible for the excessive cleavage of OPA1 in p32^−/−^ MEFs, we compared the proteolytic processing pattern of OPA1 in wild type, YMEL1^−/−^OMA1^−/−^, YMEL1^−/−^, OMA1^−/−^, and p32^−/−^ MEFs. YMEL1^−/−^ MEFs showed a similar proteolytic processing pattern (the increase of c-, e-forms) of OPA1 as seen in p32^−/−^ MEFs (Fig. 4a). Consistent with previous reports^26, 27, 30, 33^, the c- and e-form of OPA1 completely disappeared in OMA1^−/−^ MEFs. OPA1 can be processed at two protease cleavage sites, S1 (cleaved by OMA1: generates c-, e-form) and S2 (cleaved by YME1L: generates d-form) ^30^. The increase of c-, e-form of OPA1 in p32^−/−^ MEFs indicated that OMA1 is responsible for the excessive cleavage of OPA1 in p32^−/−^ MEFs. To further support OMA1-dependent proteolysis of OPA1, two independent OMA1 siRNAs were transfected into p32^−/−^ MEFs. Figure 4b and SI Fig. S3a shows that both OMA1 siRNAs successfully reversed the excessive cleavage of OPA1 in p32^−/−^ MEFs. We also demonstrated that p32 knockdown did not cause the excessive proteolytic cleavage of OPA1 in OMA1^−/−^ MEFs, further suggesting that OMA1 is a critical downstream mitochondrial protease of p32 (Fig. 4c, SI Fig. S3b). Because OMA1 activation is associated with its auto-catalytic degradation^28^, the OMA1 protein level decreased in p32^−/−^ MEFs due to its activation. However, p32 did not interact with YME1L, OPA1, or OMA1 (SI Fig. S4).

### Mitochondrial energy production is important to protect OPA1 from proteolytic cleavage

It was reported that mitochondrial membrane potential and ROS control the activity of OMA1 for cleavage of OPA1^28, 29^. We tested whether p32 expression controls the mitochondrial ROS or membrane potential, by staining the wild type and p32^−/−^ MEFs with MitoSOX and TMRE for mitochondrial ROS, and membrane potential, respectively. Under normal growth conditions, we observed no significant change in the level of mitochondrial ROS in p32^−/−^ MEFs (SI Fig. S5a), and supporting this conclusion there was no increase in the oxidized protein population in mitochondria of p32^−/−^ MEFs (SI Fig. S5b lane 4), but the total and cytosolic oxidized proteins increased in p32^−/−^ MEFs (SI Fig. S5b lane 2 and lane 6). Since oxidized proteins in the cytosolic fraction increased in p32^−/−^ MEFs, the role ROS in the proteolytic cleavage of OPA1 in p32^−/−^ MEFs cannot be excluded. The role of ROS was evaluated in the wild type and p32^−/−^ MEFs after treatment with H_2_O_2_ or a series of ROS scavengers, *N*-acetyl-l-cysteine (NAC), alpha-lipoic acid (ALA), and l-glutathione reduced (GSR). None of the tested ROS scavengers reversed the excessive proteolytic cleavage of OPA1 in p32^−/−^ MEFs, and H_2_O_2_ treatment showed a minimal effect (SI Fig. S5c, d), indicating that ROS are not a critical regulator of the proteolytic cleavage of OPA1 in p32^−/−^ MEFs.

**Figure 5:**
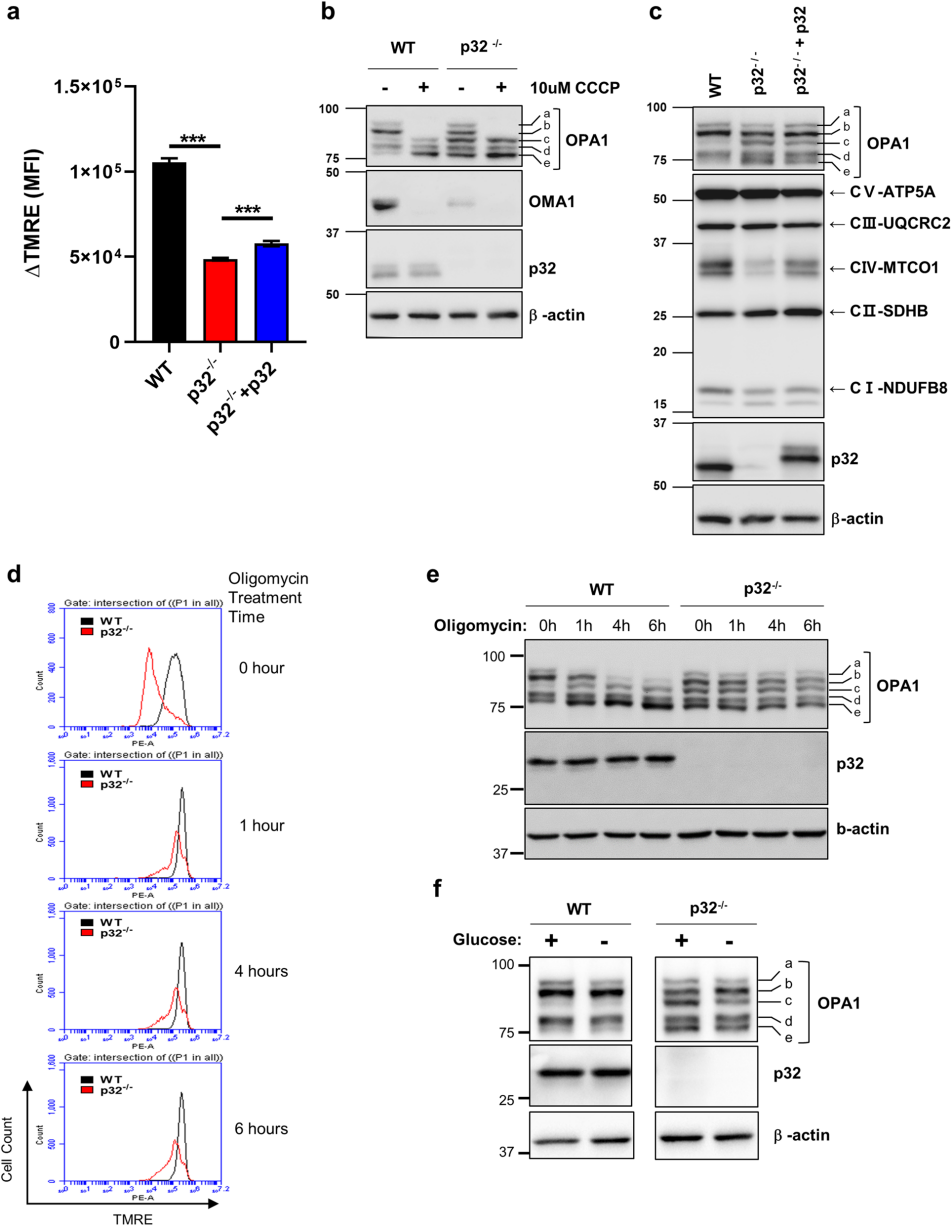
Mitochondrial membrane potential was reduced in p32^−/−^ cells and led to proteolytic processing of OPA1. (**a**) Wild type, p32^−/−^ and p32^−/−^ + p32 MEFs were incubated with/without 50 μM CCCP for 1 h, stained with TMRE and subjected to FACS analysis to measure mitochondrial membrane potential by the change of mean fluosecence intensity (MFI) after 50 μM CCCP treatment (ΔTMRE). Averages of ΔTMRE from 3 independent experiments were plotted with SD as error bar. Student’s t test, ***p < 0.001. (**b**) Wild type and p32^−/−^ MEFs were treated with 0 or 20 μM CCCP for 1 h and subjected to western blotting for indicated proteins. (**c**) Wild type and a p32^−/−^ MEFs were transfected with a p32 expression vector as indicated and subjected to western analysis for indicated proteins. (**d**) Wild type and a p32^−/−^ MEFs were treated with 1 μM oligomycin for the indicated time points, stained with TMRE and subjected to FACS analysis. (**e**) Wild type and a p32^−/−^ MEFs were treated with 1 μM oligomycin for indicated time and subjected to western blotting for indicated proteins. (**f**) Wild type and a p32^−/−^ MEFs were cultured with or without glucose for 24 h and subjected to western blotting for indicated proteins. Western blot images were cropped from the same gel.

In contrast, the mitochondrial membrane potential decreased significantly in p32^−/−^ MEFs (Fig. 5a, SI Fig. S6), and statistical analysis from four independent experiments showed about 55% reduction in mitochondrial membrane potential in p32^−/−^ MEFs compared to that in wild type MEFs (Fig. 5a). Ectopic expression of p32 in p32^−/−^ MEFs partially restored membrane potential (Fig. 5a). To examine the role of mitochondrial membrane potential on the proteolytic cleavage of OPA1, carbonyl cyanide m-chlorophenylhydrazone (CCCP) was used to abolish mitochondrial membrane potential. As shown in Fig. 5b, CCCP treatment induced the proteolytic cleavage of OPA1 in both wild type and p32^−/−^ MEFs. Because OMA1 activation is associated with its auto-catalytic degradation^28^, the OMA1 protein level decreased in p32^−/−^ MEFs and CCCP treated cells due to its activation. Since mitochondrial membrane potential is generated by an electron transport system (ETS), the protein levels of key ETS components were analyzed by Western blotting. Figure 5c shows a reduction of mitochondrially encoded cytochrome c oxidase I (MTCO1), and NADH: ubiquinone oxidoreductase subunit B8 (NDUFB8) in p32^−/−^ MEFs. Importantly, the protein level of MTCO1 was restored successfully by the re-expression of p32 (Fig. 5c lane 3). Similar results were seen for OPA1^−/−^ and OPA1^−/−^+OPA1 MEFs (SI Fig. S7a). However, protein levels of MTCO1 and NDUFB8 of p32^−/−^ MEFs were not recovered by OMA1 knockdown despite the restoration of functional uncleaved OPA1 (SI Fig. S7b). To further test if mitochondrial membrane potential controls the proteolytic processing of OPA1, we used oligomycin to elevate mitochondrial membrane potential. As shown in Fig. 5d, mitochondrial membrane potential was successfully elevated in both wild type and p32^−/−^ MEFs. As a surprise, oligomycin treatment enhanced proteolytic processing of OPA1 in wild type despite the elevation of mitochondrial membrane potential (Fig. 5d,e). Interestingly, p32^−/−^ MEFs did not show the severe proteolytic cleavage of OPA1 in response to oligomycin treatment (Fig. 5e). Since oligomycin inhibits mitochondrial ATP synthase, mitochondrial energy generation is completely blocked with extremely high mitochondrial membrane potential. In contrast, glucose starvation activates metabolic reprogramming that elevates energy production from mitochondria. As shown in Fig. 5f right panel, glucose starvation partially restored the proteolytic processing pattern of OPA1 (increased b-form and decreased c-form) in p32^−/−^ MEFs. In contrast, the proteolytic processing pattern of OPA1 in wild type MEFs was not changed by glucose starvation (Fig. 5f left panel).

### p32/C1QBP deficiency sensitizes mitochondria to metabolic stress

**Figure 6:**
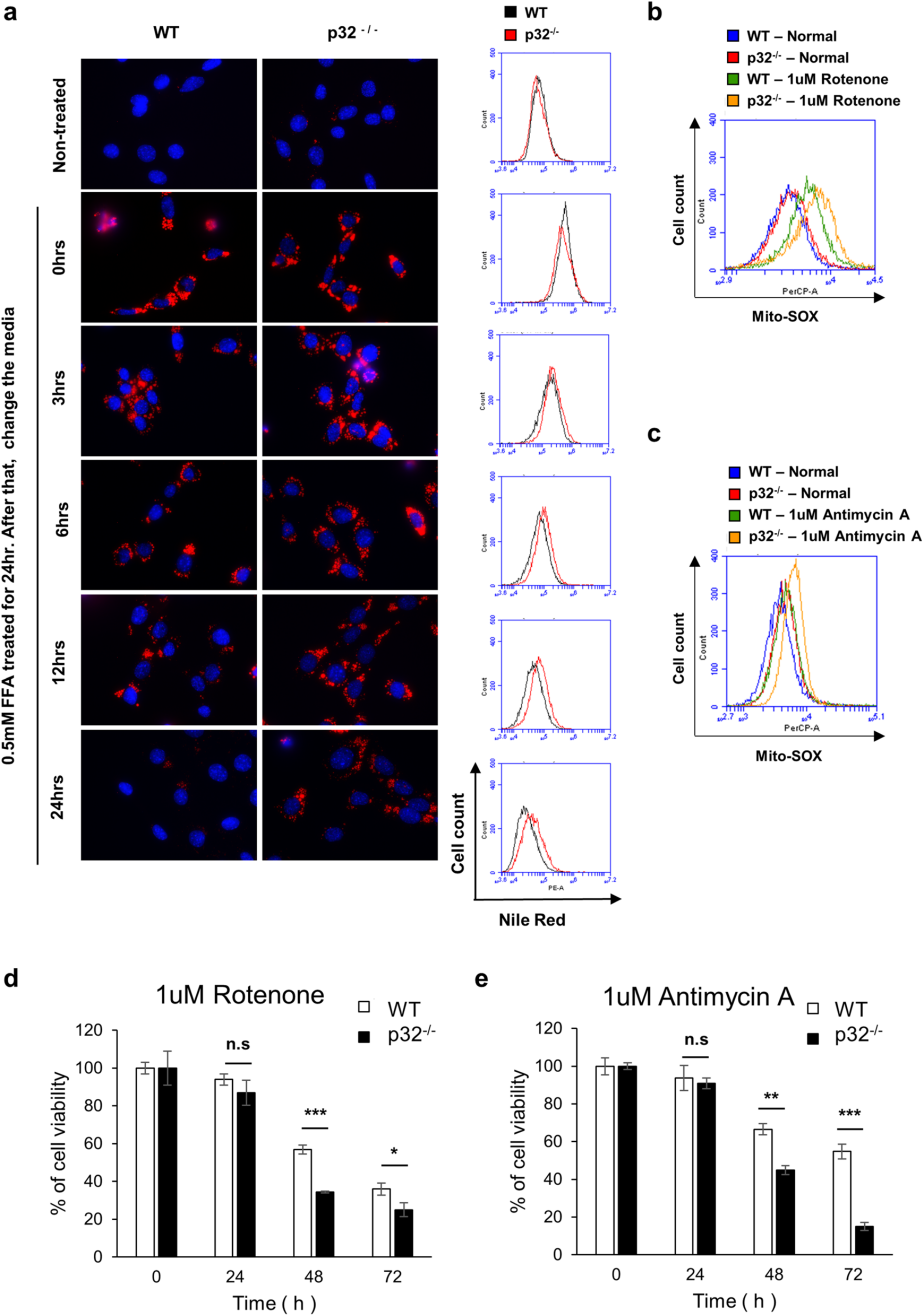
Genetic ablation of p32/C1QBP causes decreased lipid utilization, increased stress-induced mitochondrial ROS production and correlated with cell viability. (**a**) Wild type and p32^−/−^ MEFs were treated with 0.5 mM FFA for 24 h to measure the accumulation of lipid droplets. The cell culture media were changed to normal media, incubated for the indicated amount of times (0, 3, 6, 12, 24 h) and subjected to Nile red staining to visualize lipid droplets. Representative fluorescent microscopy images of Nile red staining were presented in the left panel. Scale bar 20 μm. FACS histogram of the same samples was presented in the right panel. (**b**,**c**) Wild type and p32^−/−^ MEFs treated with 0 or 1 μM rotenone or 1 μM antimycin A for 24 h, stained with MitoSox and subjected to FACS analysis. Representative FACS histogram is presented. (**d**,**e**) Wild type and p32^−/−^ MEFs were treated with 1 μM rotenone or 1 μM antimycin A for indicated time and subjected to MTT assay. % of cell viability plotted with SD as error bars. Student’s t test, *p < 0.05, *n.s.* non-significant.

The functional integrity of mitochondria was tested by lipid utilization assay. Wild type and p32^−/−^ MEFs were treated with 0.5 mM free-fatty acid (FFA) and chased the amount of lipid by Nile red staining over time to determine the catabolic rate. Figure 6a and SI Fig. S8 showed a decrease in the catabolic rate of lipid in p32^−/−^ MEFs, possibly due to defects in mitochondrial β-oxidation. In addition, wild type and p32^−/−^ MEFs were treated with rotenone (ETS complex I inhibitor), antimycin A (ETS complex III inhibitor), and malonic acid (ETS complex II inhibitor). Interestingly, both rotenone and antimycin A treatment induced higher mitochondrial ROS generation in p32^−/−^ MEFs than in wild type (Fig. 6b,c), and significantly reduced the cell viability (Fig. 6d,e). In contrast, malonic acid treatment did not induce mitochondrial ROS generation and toxicity (SI Fig. S9). Together, these results suggested that mitochondria in p32^−/−^ were more susceptible to mitochondrial metabolic stress.

### The genetic ablation of p32/C1QBP induces a glycolytic metabolic shift and causes glucose addiction

**Figure 7:**
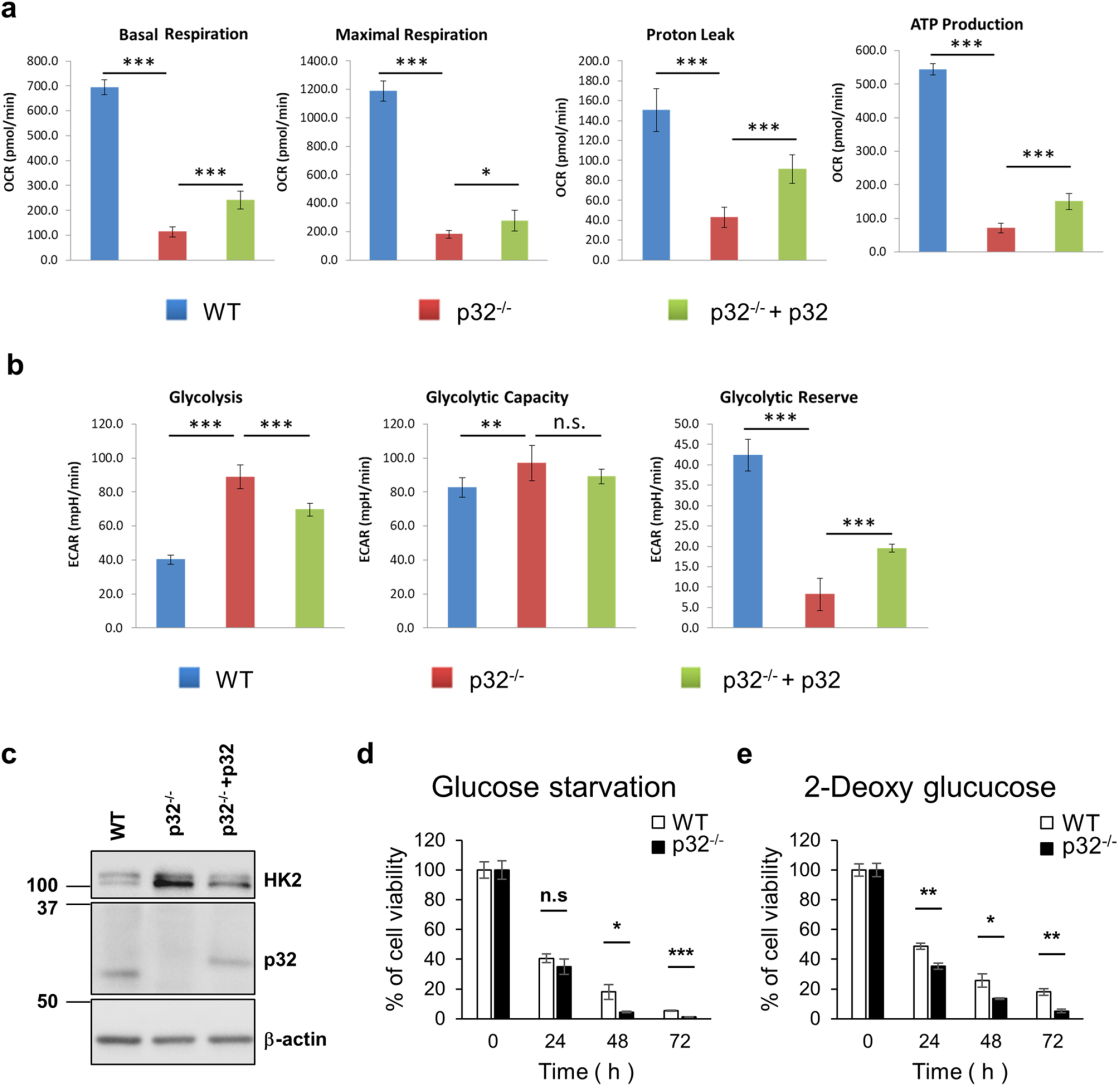
The metabolic shift from OXPHOS to glycolysis was induced by genetic ablation of p32/C1QBP resulting in glucose addiction. (**a**) Wild type, p32^−/−^ and p32^−/−^ + p32 MEFs were subjected to Agilent Seahorse XF Cell Mito Stress Test to measure basal respiration, maximal respiration, proton leak and mitochondrial ATP production. Bar graphs illustrate the absolute value of OCR that was calculated from SI Fig S8a,b and plotted with SD as error bars. Student’s t test, *p < 0.05, ***p < 0.001. (**b**) Wild type, p32^−/−^ and p32^−/−^+p32 MEFs were subjected to Agilent Seahorse XF glycolysis stress test to measure glycolysis, glycolytic capacity and glycolytic reserve. Bar graphs illustrate the absolute value of ECAR that was calculated from SI Fig S8c,d and plotted with SD as error bars. Student’s t test, **p < 0.01, ***p < 0.001, *n.s*. non-significant. (**c**) Wild type and p32^−/−^ MEFs were transfected with pcDNA or a p32 expression vector as indicated and subjected to Western analysis for hexokinase 2 (HK2), p32 and β-actin. (**d**) Wild type and p32^−/−^ MEFs were incubated in complete glucose-free medium for the indicated time and subjected to MTT assay. % of cell viability shown with SD as error bars. Student’s t test, *p < 0.05, ***p < 0.001, *n.s*. non-significant. (**e**) Wild type and p32^−/−^ MEFs were treated with 5 mM 2-deoxy-d-glucose for the indicated time and subjected to MTT assay. % of cell viability shown with SD as error bars. Student’s t test, *p < 0.05, ***p < 0.001, *n.s*. non-significant.

OXPHOS in mitochondria and glycolysis in the cytosol are two critical metabolic pathways that generate cellular energy ATP. The role of p32 in cellular energy metabolism was investigated by measuring the oxygen consumption rate (OCR), an indicator of mitochondrial OXPHOS, and the extracellular acidification rate (ECAR), an index of lactate production and glycolysis. Wild type, p32^−/−^ and p32^−/−^+p32 MEFs were used to show OCR changes in response to uncoupling OXPHOS from ATP synthesis by using trifluoromethoxy carbonyl cyanide phenylhydrazone (FCCP) to measure maximum OCR, and antimycin-A/rotenone inhibition of the electron transport chain (ETC) to measure non-mitochondrial respiration (SI Fig. S10a). The basal, maximal respiration, proton leak, and ATP production were calculated as indicated in SI Fig. S10b. As shown in Fig. 7a, p32^−/−^ MEFs showed a significant reduction of basal, maximal respiration, proton leak and ATP production, which was partially restored by ectopic expression of p32. We also performed glycolysis stress test with wild type, p32^−/−^ and p32^−/−^+p32 MEFs (SI Fig.S10c) to measure glycolysis, glycolytic capacity and glycolytic reserve as indicated in SI Fig. S10d. In p32^−/−^ MEFs, the amount of glycolysis was increased up to maximum capacity reducing glycolytic reserve which was partially reversed by ectopic expression of p32 (Fig. 7b). These results suggested that the genetic abolition of p32 triggers a glycolytic metabolic shift.

Hexokinase 2 (HK2) catalyzes the rate-limiting and first obligatory step of glycolysis, which is the ATP-dependent phosphorylation of glucose to glucose 6-phosphate^34^. Thus, we tested if p32 expression could regulate the expression of HK2. HK2 protein level increased in p32^−/−^ MEFs and reversed by restoring p32 expression (Fig. 7c). Together, these results suggest that genetic deletion of p32 abolishes mitochondrial energy production and increased HK2 (a rate-limiting enzyme for glycolysis) expression resulting in a glycolytic metabolic shift.

Without p32 expression, cellular energy metabolism would rely more on glycolysis due to defective mitochondrial respiration and would lead to glucose addiction. Hence, wild type and p32^−/−^ MEFs were treated with glucose-depleted media or 2-DG, an inhibitor of glycolysis, and cell viability measured over 3 days. Figure 7d,e show that p32^−/−^ MEFs were more sensitive to glucose deprivation and 2-DG treatment, suggesting that p32^−/−^ MEFs were more addicted to glucose than wild type.

### Genetic ablation of p32/C1QBP induces apoptosis and inhibits spheroid formation

**Figure 8:**
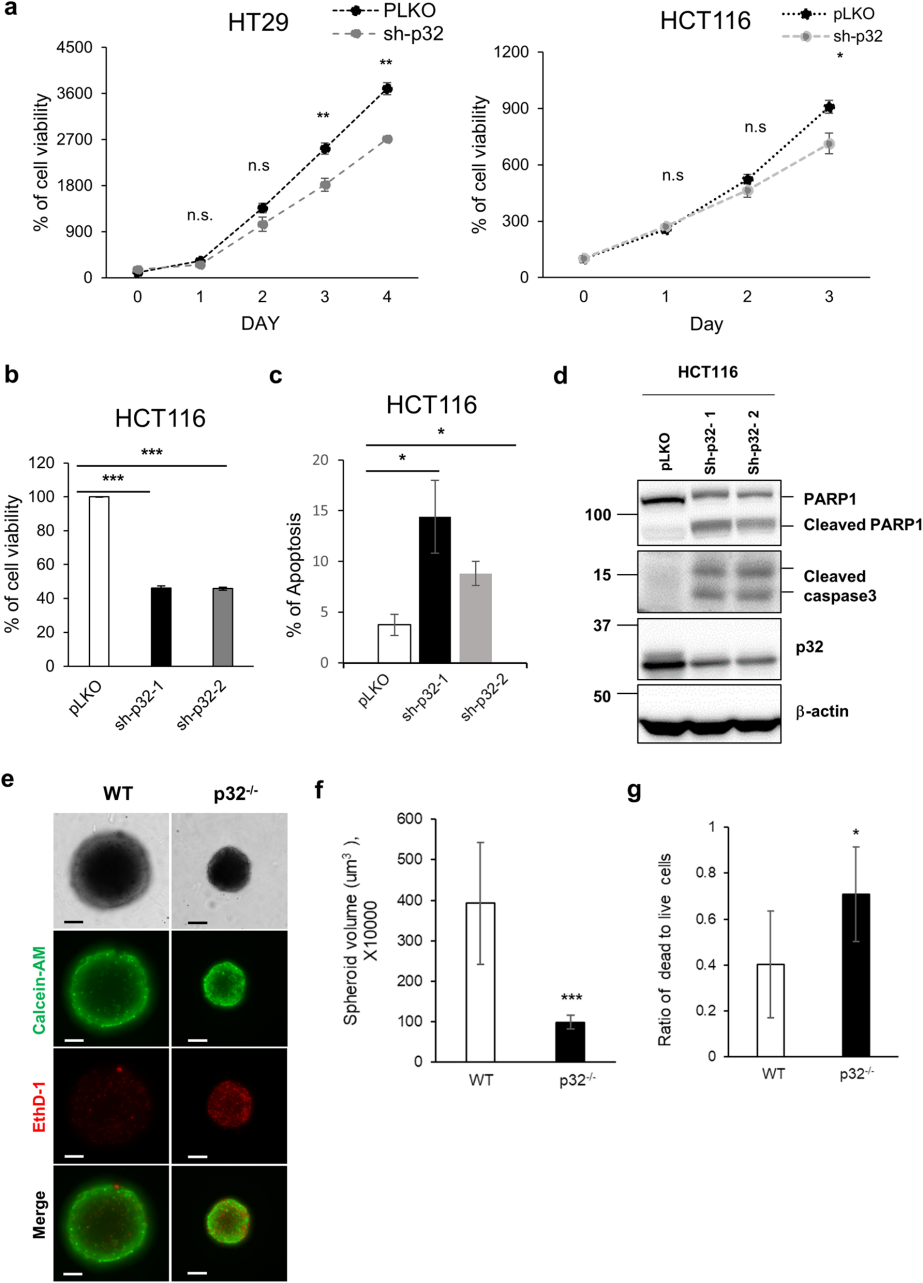
p32/C1QBP deficiency induced apoptosis and inhibited the spheroid formation. (**a**) HT29 and HCT116 cells were infected with pLKO or sh-p32 lentivirus, selected for 1 week with puromycin to generate stable knockdown cells. The cells incubated with normal growth media for the indicated time and subjected to MTT assay. % of cell viability plotted with SD as error bars. Student’s t test, *p < 0.05, **p < 0.01, *n.s.* non-significant. (**b**) HCT116 cells were infected with pLKO or two independent sh-p32 lentiviruses, selected for 1 week with puromycin to generate stable knockdown cells. HCT116-pLKO, -sh-p32-1 and -sh-p32-2 cells were incubated with normal growth media for 3 days and subjected to MTT assay. % of cell viability with SD as error bars shown. Student’s t test, ***p < 0.001. (**c**) The FACS plots of apoptotic cell analysis. HCT116-pLKO and two independent HCT116-sh-p32 cells were stained with Annexin-V/PI and analyzed by FACS. Averages of 3 independent experiments were presented with SD as error bars. Student’s t test, *p < 0.05. (**d**) HCT116-pLKO and two independent HCT116-sh-p32 cells were subjected to Western blotting for indicated proteins. (**e**) Representative microscopy pictures of spheroids generated by wild type and p32^−/−^ MEFs. Spheroids were stained with calcein-AM for live cells and EthD-1 for dead cells. Scale bar 100 μm. (**f**) The average size of 8 independent spheroids from wild type and p32^−/−^ MEFs was measured and graphed. Error bars are SD and Student’s t test, *p < 0.05, ***p < 0.001, *n.s.* non-significant. (**g**) Dead to live cells ratio of 6 independent spheroids from wild type and p32^−/−^ MEFs was measured and graphed. Error bars are SD and Student’s t test, *p < 0.05, ***p < 0.001, *n.s.* non-significant.

The role of p32 in tumor growth was investigated by the stable knockdown of p32 in two colorectal cancer cell lines, HT29 and HCT116, and the growth rate was measured by MTT assay. The growth rates of both HT29 and HCT116 cells were reduced significantly by p32 knockdown (Fig. 8a). This conclusion was supported further by knockdown experiments of p32 in HCT116 cells using two independent shRNAs (Fig. 8b). The control and p32 knockdown HCT116 cells were seeded at the same cell density (1 × 10^4^/well), and the cell viability checked using MTT assay 3-days after seeding. Figure 8b shows a decrease in the cell viability in two independent p32 knockdown HCT116 cells suggesting cell cycle delay or cell death. Since the cell cycle is not affected by p32 expression (data not shown), we checked for annexin-V positive apoptotic cells to monitor the apoptotic cell death by FACS analysis. Notably, two independent p32 knockdown HCT116 cells (sh-p32-1 and sh-p32-2) showed an increase in annexin-V positive apoptotic cells compared with pLKO (empty vector) control cells (Fig. 8c, SI Fig.S11). In addition, p32 knockdown HCT116 cells showed an increase in active caspase 3 and cleaved poly (ADP-ribose) polymerase 1 (PARP1), hallmarks of apoptosis, suggesting apoptosis induction by inhibiting p32 expression in HCT116 cells (Fig. 8d).

The glycolytic metabolic shift has been linked extensively to the hypoxic microenvironment of 3D-spheroid^35^. The role of p32 in the formation of a 3-D spheroid was tested through the spheroid formation assay using wild type and p32^−/−^ MEFs. Figure 8e,f show a significant reduction in the size of p32^−/−^ spheroids compared to wild type spheroids, suggesting that p32 depletion inhibits spheroid formation. The level of HIF-1α was increased in wild type spheroid, probably due to its bigger size than p32^−/−^ spheroid limiting oxygen accessibility (SI Fig. S12a). In monolayer, the level of HIF-1α was similar between wild type and p32^−/−^ MEFs (SI Fig. S12b). Unlike HCT116 colon cancer cells, p32^−/−^ MEFs did not show the signature of apoptosis, such as cleaved PARP1 or activated caspase in monolayer culture (SI Fig. S13). In contrast, the spheroid of p32^−/−^ MEFs contains a lot of dead cells (labeled with Ethidium homodimer-1, EthD-1) indicating p32 plays a role in cell survival under 3D-spheroid forming condition (Fig. 8e,g).

## Discussion

The mitochondrion is a central organelle for cellular metabolism, including OXPHOS, the tricarboxylic acid cycle (TCA cycle), β-oxidation of fatty acids, calcium signaling, and heme biosynthesis^36^. Therefore, mitochondrial dysfunction has been linked extensively with cancer, neurodegenerative diseases, metabolic diseases, and aging^36, 37^. Mitochondria maintain their function through dynamic behaviors, such as fusion, fission, transport, and mitophagy^36^. The results of this study showed that the genetic ablation of p32 leads to mitochondrial fragmentation and swelling (Figs. 1, 2), emphasizing the requirement of p32 for the maintenance of mitochondrial morphology and probably their function. As expected, we noted several features of mitochondrial dysfunction in p32^−/−^ cells such as decreased mitochondrial membrane potential (Fig. 5a), decreased levels of ETS complex proteins (Fig. 5c), and decreased lipid utilization (linked to mitochondrial β-oxidation of fatty acids, Fig. 6a). Considering that p32 is essential for mitochondrial translation^9^, it is possible that the mitochondrial defects in p32^−/−^ cells are due to the failure of mitochondrial translation, and the results from the p32-dependent MTCO1 protein expression support this. However, the role of the p32-dependent regulation of mitochondrial morphology in the maintenance of mitochondrial function could not be excluded.

Although p32 regulates mitochondrial morphology in neuronal cells by promoting Parkin degradation, it is not clear if it regulates mitochondrial morphology in cells that do not express Parkin. Parkin is not present in MEFs (SI Fig. S1f) and most cancer cells^38, 39^, and the p32-dependent regulation of mitochondrial morphology could not be associated with Parkin. Instead, p32 controls the OMA1-dependent proteolytic processing of OPA1 (Figs. 3, 4), and to identify the mechanism, we examined the physical interaction between p32, OPA1, and OMA1. However, p32 did not interact with OPA1 or OMA1 (SI Fig. S4). Because OMA1 activation occurs in response to mitochondrial stress such as depolarization of the mitochondrial membrane and ROS^28, 29, 40, 41^, it is possible that p32 controls membrane potential or mitochondrial ROS to regulate the proteolytic activity of OMA1. To this end, we showed that the genetic ablation of p32 decreased mitochondrial membrane potential but did not affect the mitochondrial ROS production under normal growth conditions (Fig. 5a, SI Fig. S5a,b). Consistently, cytoplasmic and mitochondrial ROS scavengers did not affect OPA1 proteolytic processing in p32^−/−^ MEFs (SI Fig. S5c,d) but CCCP (mitochondrial uncoupler) treatment strongly induced the proteolysis of OPA1 in both wild type and p32^−/−^ MEFs (Fig. 5b). Because OMA1 activation is associated with its auto-catalytic degradation^28^, the OMA1 protein level decreased in p32^−/−^ MEFs and CCCP treated cells due to its activation (Figs. 4, 5b). Interestingly, oligomycin induces the proteolytic cleavage of OPA1 in wild type MEFs despite the fact that mitochondrial membrane potential was induced (Fig. 5e). Since oligomycin inhibits mitochondrial ATP synthase, mitochondrial energy generation is completely blocked with extremely high mitochondrial membrane potential. Glucose starvation could induce mitochondrial ATP generation to compensate for the reduced amount of cytosolic ATP generation (glycolysis). Thus, we used glucose starvation to elevate mitochondrial ATP generation in wild type and p32^−/−^ MEFs. As shown in a Fig. 5f, glucose starvation did partially inhibit proteolytic cleavage of OPA1 in p32^−/−^ MEFs. In contrast, wild type MEFs showed no change in OPA1. These results suggested that mitochondrial energy (ATP) production would also play a role to regulate proteolytic cleavage of OPA1 as well as mitochondrial membrane potential.

To examine if p32 is essential for the maintenance of mitochondrial function, we measured lipid utilization and stress-induced mitochondrial ROS production in wild type and p32^−/−^ MEFs. As expected, the rate of lipid utilization decreased in p32^−/−^ MEFs probably due to impairment of mitochondrial β-oxidation (Fig. 6a). Interestingly, ETS complex I and III inhibitors (rotenone and antimycin A, respectively) induced more mitochondrial ROS production in p32^−/−^ MEFs than in wild type and reduced cell viability (Fig. 6b–e). In contrast, ETS complex II inhibitor (malonic acid) did not affect mitochondrial ROS production and cell viability. Because complex II activity depends on nuclear-encoded succinate dehydrogenase (SDH), but the activities of complexes I and III depend on both mitochondrial and nuclear-encoded genes^42^, it would be reasonable to surmise that mitochondrial p32 does not affect cellular responses to nucleus-encoded complex II inhibition.

The metabolic shift from OXPHOS to glycolysis due to genetic ablation of p32 has been reported in breast cancer cells^10^ and dendritic cells^43^, and similarly the p32^−/−^ MEFs showed a glycolytic metabolic shift (Fig. 7a,b). We further demonstrated that p32 negatively regulates the expression of hexokinase II, a key rate-limiting mitochondrial-associated enzyme in glycolysis. (Fig. 7c). The glycolytic shift of energy metabolism in p32^−/−^ MEFs decreased cell viability upon glucose starvation or glycolysis inhibition, suggesting that p32 inhibition induced glucose addiction (Fig. 7d,e).

Most cancer cells rely avidly on glycolysis, even when oxygen is sufficient for OXPHOS (so-called aerobic glycolysis or the ‘Warburg effect’). Although glycolysis is studied extensively as a potential therapeutic target for various cancers, mitochondrial function is also essential for cancer cell survival due to essential metabolic pathways in mitochondria including pyrimidine synthesis, lipid synthesis, heme synthesis, and fatty acid oxidation^44^. Therefore, mitochondria are considered as metabolic sensing switch to trigger apoptosis in response to stress^44^. We and others observed that the genetic ablation of p32 inhibits the growth of monolayer cancer cells (Fig. 8a,b), 3D spheroids (Fig. 8e), and xenograft tumors^10^. Furthermore, we showed that knockdown of p32 could induce significant apoptotic cell death in monolayer colon cancer cells (Fig. 8c,d). Higher expression of p32 has been related to poor clinical outcomes in breast^15, 45^, ovarian^13^, endometrial^46^, cervical^47^, and colon^17^ cancers. These data support the critical role p32 plays in the survival of various cancer cells.

Overall, the results of this study showed the critical role of p32 in protecting mitochondria from fragmentation and swelling by inhibiting OMA1-dependent proteolytic processing of OPA1. Genetic ablation of p32 abolished mitochondrial structure and function, sensitized cells to mitochondrial stress and triggered a metabolic shift from OXPHOS to glycolysis, features consistent with the glucose addiction and apoptosis of cancer cells. Our results provide a unique perspective for understanding the role of p32 in cellular metabolism, with the potential to unravel new therapeutic strategies for cancer treatment.

## Materials and methods

### Cell and culture condition

Wild type and p32 knockout primary mouse embryo fibroblasts (MEFs) were generously provided by Dr. Dongchon Kang^43^. OMA1^−/−^, YME1L^−/−^, OMA1^−/−^YME1L^−/−^, MEFs were generously provided by Dr. Thomas Langer^30^. OPA1^−/−^ and OPA1^−/−^+OPA1 MEFs were generously provided by Dr. David Chan^48^. Cells maintained in Dulbecco's modification of Eagle medium (DMEM; Hyclone SH30243.01) supplemented with 10% (v/v) fetal bovine serum (Gibco 12483-020) and 1% (v/v) antibiotic–antimycotic 100× (Gibco 15240-062) at 37 °C in 5% CO_2_. Human embryonic kidney 293 cells (293T) were used for transfection and virus production and maintained in DMEM supplemented with 10% FBS and 1% antibiotics. All cell lines were plated on a 100 mm plate.

### Antibodies

Antibodies against the following proteins were used: p32 (Santa Cruz Biotechnology sc-4879), OPA1 (BD Biosciences 612607), OMA1 (Santa Cruz Biotechnology sc-515788), YME1L (Proteintech 11510-1-AP), Mfn1 (Santa Cruz Biotechnology sc-166644), Mfn2 (Santa Cruz Biotechnology sc-100560), Drp1 (Santa Cruz Biotechnology sc-32898), TOM20 (Santa Cruz Biotechnology sc-11415), Hexokinase2 (Santa Cruz Biotechnology sc-374091), Total OXPHOS Rodent WB Antibody Cocktail (Abcam ab110413), E-cadherin (Cell Signaling Technology 3195T), PARP (Cell Signaling Technology 9532S), Cleaved Caspase-3 (Cell Signaling Technology 9661) and β-actin (Santa Cruz Biotechnology sc-47778).

### Fluorescence microscopy

The cells were fixed with 4% paraformaldehyde at room temperature for 15 min and subsequently permeabilized in 0.2% Triton X-100 (Sigma-Aldrich X100) in PBS for 5 min. The cells were blocked with PBS containing 5% normal goat serum and 0.1% Triton X-100 for 20 min. The cells were incubated with primary antibody (Tom20-specific antibody, Santa Cruz Biotech. SC-11415) at 4 °C for overnight. After washing with PBS containing 0.1% Triton X-100, the cells were incubated with secondary antibody (Rhodamine Red-X AffiniPure Goat Anti-Rabbit IgG (H+L), Jackson ImmunoResearch Laboratories 111-295-144) for 1 h. DAPI Fluoromount-G Mounting Medium (Southern Biotech 0100-20) was used to counterstain the nuclei. All images were obtained with a fluorescence microscope (Olympus BX53, Shinjuku, Tokyo, Japan).

### Mitochondrial fusion assay

ZEISS LSM 880 confocal microscope with Airyscan in Center for Research Facility in Chungnam National University was used for the mitochondrial fusion assay. 405 nm laser was used for photoconversion of PAGFPmt. Z stack images of PAGFPmt and DsRed2mt using a step size 0.5 μm were acquired using 488 nm and 561 nm laser immediately after photoactivation and after 10, 20, 30 or 40 min. Average fluorescence intensity of photoactivated PAGFPmt were measured by ZEISS ZEN 2.3 software.

### Transmission electron microscopy

The cells were initially fixed for 2 h with 2.5% glutaraldehyde in 0.1 M phosphate (pH 7.3) at room temperature. After this fixation, the tissues were treated with 1% OsO4 plus 1.5% potassium ferrocyanide in 0.1 M phosphatete buffer (pH 7.3) for 1 h at 4 °C in the dark and embedded in Epon 812 after dehydration in an ethanol and propylene oxide series. Polymerization was conducted using pure resin at 70 °C for 24 h. Ultrathin sections (70 nm) were obtained with an ultramicrotome (Leica EM UC7, Leica, Austria), which were then collected on 100-mesh copper grids. After staining with 2% uranyl acetate (15 min) and lead citrate (5 min), the sections were examined by transmission electron microcopy at 120 kV (Technai G2 Spirit Twin, Thermo Fisher Scientific, USA).

### Immunoblotting

The cells were lysed in NETN buffer (NP-40, 0.5 mM EDTA, 20 mM Tris and 150 mM NaCl) containing protease inhibitor cocktail (Sigma-Aldrich P8340), phosphatase inhibitor cocktail (Sigma-Aldrich P0044) and 1 m M dithiothreitol (DTT) for 15 min on ice. Lysates were cleared by centrifugation at 15,000 rpm at 4 °C for 15 min and supernatants were performed to Sodium dodecyl sulfate polyacrylamide gel electrophoresis (SDS-PAGE). The primary antibody dilution for western blot was 1:1,000 in 2% BSA. HRP-conjugated anti-Rabbit IgG (Sigma-Aldrich A0545) or anti-mouse IgG (Sigma-Aldrich A9917) were used for protein development. The protein signal intensity of detected protein was analyzed using Fusion Solo ChemiDOC system (Fisher biotech, Australia) and Bio1D software (Fisher biotech, Australia).

### Electroporation

Neon Transfection System (Invitrogen MPK10096) was used for electroporation, according to the manufacturer’s protocol. MEFs (5 × 10^5^ cells) were transfected with 5 μg of plasmid using microporator (1,350 V for 15 ms in 2 pulses) and incubated in DMEM without antibiotics for 24–48 h.

### TMRE staining

Mitochondrial membrane potential was determined by Tetramethylrhodamine, Ethyl Ester Perchlorate (TMRE: Invitrogen T669). Cells (3 × 10^5^/well) were seeded in 6-well plate and treated with/without 50 μM CCCP for 1 h and stained with 20 nM TMRE for 20 min at 37 °C incubator. The stained cells were harvested and analyzed by BD Acuri C6 Plus (BD Biosciences USA San Jose, CA, USA).

### Nile Red staining

Lipid accumulation was determined by Nile Red (Santa Cruz biotechnology sc-203747). Cells (3 × 10^5^ cells/well) were seeded in 6-well plate and treated with 0.5 mM FFA for 24 h. Cells were harvested and diluted 1 × 10^6^ cells/ml. The diluted cells were stained with 0.125 μg/ml of Nile Red for 10 min at a 37℃ incubator. The stained cells were analyzed by BD Acuri C6 Plus (USA San Jose, CA, USA).

### MtioSOX staining

Mitochondrial Reactive oxygen species was determined by 5 mM Mito-SOX (Invitrogen M36008). Cells (3 × 10^5^/well) were seeded in 6-well plate and treated 2 μl/well Mito-SOX at a 37 °C for 30 min. The stained cells were harvested and analyzed by BD Acuri C6 Plus (USA San Jose, CA, USA).

### Annexin V staining

Cell apoptosis was determined by Annexin V, FITC conjugate kit (BD Pharmingen 556547). Cells were harvested and diluted 1 × 10^6^ cells/ml. The diluted cells were stained with Annexin V, FITC conjugate kit according to the manufacturer's protocol. The stained cells were analyzed by BD Acuri C6 Plus (USA San Jose, CA, USA).

### Formation of cell spheroids

Spheroids were formed in the 96-well Black/Clear Round Bottom plate (Corning 4515) for 6 days. Cells (8 × 10^4^ cells/well) were seeded and changed the media every 2 days by gently pipetting. Cultured cells were evaluated microscopically each day and were determined the spheroid volume (μm^3^) by diameter determination.

### Cell viability assay in spheroids

LIVE/DEAD Viability/Cytotoxicity Kit (Invitrogen L3224) was used to determine live and dead cells in the spheroid. Cultured spheroids were stained with 2 μM calcein-AM and 4 μM Ethidium homodimer-1 (EthD-1) incubated simultaneously at 37 °C for 1 h. Analysis of labeled cells was performed using Cell Insight CX7 LZR high-content screening (HCS) Platform (Thermo Scientific USA, MA, USA).

### Mitochondrial bioenergetics profiling by seahorse bioanalyzer

The cells were seeded at a density of 3 × 10^4^ cells/well on a XF24 culture plate. DMEM media was contained 2 mM glutamine, 1 mM pyruvate and 25 mM glucose in base media. Glycolytic flux media was contained 2 mM glutamine in base DMEM media. During the calibration of the sensor cartridge, the cells were washed with assay media and incubated at 37 °C without CO_2_ for 1 h before measurement. Oxygen consumption rate (OCR) and extracellular acidification rate (ECAR) measurements were performed using an XF24 Extracellular Flux analyzer (Seahorse Bioscience, North Billerica, MA, USA) as described.

## Supplementary information

Supplementary file1 (PDF 6131 kb)
