# Supplementary material for: p32/C1QBP regulates OMA1-dependent proteolytic processing of OPA1 to maintain mitochondrial connectivity related to mitochondrial dysfunction and apoptosis
Source: Sci Rep. 2020 Jun 30;10:10618. doi: 10.1038/s41598-020-67457-w (PMC7327069; doi:10.1038/s41598-020-67457-w)

p32/C1QBP regulates OMA1-dependent proteolytic processing of OPA1 to maintain mitochondrial connectivity related to mitochondrial dysfunction and apoptosis

Running Title: p32/C1QBP regulates proteolytic activity of OMA1

Solhee Noh[1], Phorl Sophors[1], Rema Nasker[1], Oeum Kakada[1], Yuri Seo[1], Eunjung Kim[1], Hee-Seok Kweon[2], Joo-Yong Lee[1,2,#]

[1]Graduate School of Analytical Science and Technology (GRAST, Chungnam National University, Daejeon 305-764, Republic of Korea. [2]Korea Basic Science Institute, Daejeon 34133

Supplementary Information:

Supplementary Methods

Supplementary legends for Figure S1-S13

Supplementary methods

RNAi transfection

The siRNAs were purchased form Genolution, Inc. at 10 nmole scale. siRNAs were transfected into cells using Lipofectamin RNAi-MAX reagent (Invitrogen 13778-075) according to the manufacturer's instructions. The sequence of control siRNA was 5'-UUCAAUAAAUUCUUGAGGUUU-3', OMA1-1 siRNA was 5'-GGAUACAGUCAAAGUUGCAGAGUAUU-3', OMA1-2 siRNA was 5'-GCUUCUUGGUCUGAGUGCAUUUGGAUU-3' for mouse. OMA1-1 siRNA was 5'-TGGACTACTGCTTGCTGCAAAGGCT-3', OMA1-2 siRNA was 5'-TGGCAGCAAATGGAGTTCGTTGATA-3' for human. p32 siRNA was 5-GGUUGAAGAACAGGAGCCUdTdT-3′ for human.

Small/short hairpin RNA (shRNA) and lentivirus infection

To produce lentivirus expressing shRNA for p32-1 5'-CCGGGCACCAGGAGTACATTACTTTCTCGAGAAAGTAATGTACTCCTGGTGCTTTTTG-3' (TRCN00 00057106), shRNA for p32-2 5'-CCGGTCTGAATGGAAGGATACTAATCTCGAGATTAGTATCCTTCCA TTCAGATTTTTG -3' (TRCN0000370165), shRNA for p32-3 5'-CCGGCCTTGGACTGGGCCTTATATGC TCGAGCATATAAGGCCCAGTCCAAGGTTTTTG-3' (TRCN0000370164), pLKO-empty plasmids were co-transfected with the lentivirus packaging plasmids (pLP1, pLP2 and VSV-G) into 293FT cells .The virus containing cell culture media was harvested, filtered by 0.22 μm pore-size filter (GVS life sciences 7028064) and used to infect colorectal cancer cell lines (HCT116 and HT29). To generate stable p32 knockdown cells, infected cells were selected by 2 μg/ml puromycin (SIGMA P7255) for one week.

Mitochondrial morphology analysis

Mitochondrial image analysis was performed by following a publicly available method developed by McClatchey et al [1]. In the method, thresholds were chosen automatically by the Otsu algorithm[2] and the Sobel operator. Otsu algorithm was used for automatic detection of a threshold for image binarization. The Sobel operator was used to detect edges. The operator computes the computation of an approximation of the gradient of image intensity function at each image point to generate a threshold for edge detection.

Cell viability assay

Cell proliferation was determined by 3-(4,5-Dimethylthiazol-2-yl) - 2, 5 -Diphenyl tetrazolium Bromide (5 mg/ml MTT; SIGMA M5655). Cells (1x10^4/well) were seeded in 96-well plate and incubated with Malonic acid for 1~4 days. 10 μl/well MTT were added and the plate was incubated for 1~4 hours at 37°C incubator. The supernatant were removed and 100 **u**l/well dimethylsulphoxide (DMSO; SIGMA D8418) were added to solubilize the crystalize MTT. Absorbance was measured at 570 nm using a microplate reader (VersaMax, Molecular devices USA San Jose,CA).

Isolation of mitochondria

Cells were harvested and resuspended with ice-cold RSB hypo buffer. Incubation for 10 min in ice for swelling of cells. Swelling of cells transfer to 15-mL Dounce homogenizer and break the swollen cells with several strokes of the B pestle. Centrifuge the homogenate at 1300g for 5 min to pellet of nuclei, unbroken cells, and large membrane fragments. Transfer the Supernatant to new tube and repeat centrifuge until cannot see pellet. Transfer the supernatant to new 1.5 ml tube and centrifuge at 17000g for 15 min to pellet mitochondria. Supernatant is cytosol fraction and pellets is mitochondria.

Oxyblot

Cytosolic and mitochondrial fractions were prepared as previous. 2 μg of each fraction were subjected to the oxidized protein analysis. Oxidized proteins in both cytosol and mitochondrial fractions were detected using Oxyblot protein oxidation detection kit (Chemicon, S7510) according to the manufacturer's protocol.

Reactive oxygen species scavenger assay

In order to evaluate the radical scavenging effect, cells ($4x10^5$/well) were seeded in 6-well pate and incubated with different scavenger (N-Acetyl-L-Cysteine (NAC, SIGMA A9165), 50 μM L-Glutathione Reduced (GSR, SIGMA-ALDRICH G4251) and 500 μM Alpha Lipoic Acid (ALA, SIGMA T1395) or 500 μM H_2O_2 for 24 h. Treated cells were harvest and performed to immunoblotting assay for indicated proteins.

Mitochondria image analysis

A publicly available method [1] was used to analyze mitochondrial morphology in wild type, $p32^{-/-}$, and $p32^{-/-}$+-32 experimental conditions. Thirty different images per experimental condition were analyzed. We quantified average mitochondrial length, average mitochondrial width, and the ratio of width to length.

Supplementary Figure Legends

Supplementary Figure S1. p32 /C1QBP controls mitochondrial morphology

(a) Wild type, $p32^{-/-}$, and $p32^{-/-}$+p32 MEFs were fractionated into cytosol and mitochondria and subjected to Western blotting with indicated antibodies. **(b, c, d)** Computational image analysis of Fig. 1a. Thirty different images, one of each containing 3-12 cells, were analyzed. **(b)** A distribution of average mitochondrial length. **(c)** A distribution of average mitochondrial width. **(d)** A distribution of the ratio of width to length. **(e)** Representative images of mitochondrial morphology analysis. Left: original image, right: processed image, blue: nucleus, green: mitochondria, red: cytoplasm and background **(f)** Mouse brain, wild type and $p32^{-/-}$ MEFs were subjected to Western blotting for indicated proteins.

Supplementary Figure S2. Representative images of mitochondrial fusion analysis

Wild type and $p32^{-/-}$ MEFs expressing PAGFPmt and DsRed2mt were subjected to mitochondrial fusion assay. Fusion of the photo-converted fraction with the rest of the mitochondrial network dilutes activated PAGFPmt and leads to a reduction in fluorescence intensity. Images are representative z-projections at indicated time after photo-conversion. Scale bars: 10 μm.

Supplementary Figure S3. Quantitative analysis of OPA1 a-, b-, c-, d- and e-forms in Fig. 3b-c.

(a) OPA1 a-, b-, c-, d- and e-form band intensity from Fig. 3b were measured and presented as averages from 3 independent experiments with S.D. as error bars. Student's t-test, $**p<0.01$, $*p<0.05$. **(b)** OPA1 a-, b-, c-, d- and e-form band intensity from Fig. 3c were measured and presented as averages from 2 independent experiments with S.D. as error bars. Student's t-test, $**p<0.01$, $*p<0.05$.

Supplementary Figure S4. p32 /C1QBP did not interact with OPA1 or OMA1

(a) Endogenous p32 was immunoprecipitated from cell lysates of wild type and p32$^{-/-}$ MEFs and subjected to Western blotting for p32, OMA1 and OPA1. **(b)** Wild type MEFs were incubated with or without glucose for 24 hours. Endogenous OPA1 was immunoprecipitated and subjected to Western blotting for OPA1 and p32. **(c)** 293T cells were transfected with myc-tagged p32 expression plasmid. The cells were subjected to immunoprecipitation with anti-myc antibody. Total cell lysates and immunoprecipitated protein samples were subjected to Western blotting with anti-YME1L and anti-Myc antibody.

Supplementary Figure S5. ROS did not control p32/C1QBP-dependent proteolytic processing of OPA1.

(a) Wild type and p32$^{-/-}$ MEFs were stained with MitoSox and analyzed by FACS. A representative FACS histogram is presented in the left panel. Averages of MitoSox fluorescence intensity were graphed with S.D. as an error bar (right panel). Student's t-test, n.s. = non-significant. **(b)** Wild-type and p32$^{-/-}$ MEFs were fractionated into cytosolic and mitochondrial fractions and subjected to an Oxyblot assay where oxidized proteins were detected using an antibody against the dinitrophenyl moiety after the derivatization reaction. Cell lysates were subjected to Western blotting for HSP60, TOM20 (mitochondrial fraction), β-actin (cytosolic fraction) and p32. **(c)** Wild type and p32$^{-/-}$ MEFs were treated with 0, 2, 4, 8 mM NAC (N-acetyl-L-cysteine) for 24 h and subjected to immunoblot analysis for the indicated proteins. **(d)** Wild type and p32$^{-/-}$ MEFs were treated with 500 μM α-Lipoic acid (ALA), 50 μM L-glutathione reduced (GSR) or 500 μM H_2O_2 for 24 h and subjected to immunoblotting assay for indicated proteins.

Supplementary Figure S6. Mitochondrial membrane potential was reduced in p32$^{-/-}$ MEFs.

(a) Representative FACS histogram is presented in Figure 5a. **(b)** Averages of TMRM MFI (mean fluorescence intensity) from 3 independent experiments were plotted with S.D. as error bar (bottom panel). Student's t-test, ***p < 0.001.

Supplementary Figure S7. MTCO1 and NDUFB8 expression depends on OPA1 expression but OMA1 knockdown did not restore MTCO1 and NDUFB8 expression in p32$^{-/-}$ MEFs

(a) Wild type, OPA1$^{-/-}$ and OPA1$^{-/-}$+OPA1 MEFs were subjected to western blotting for indicated proteins. **(b)** Wild type and p32$^{-/-}$ MEFs were transfected with control or siRNA for OMA1 and subjected to western blotting for the indicated proteins.

Supplementary Figure S8. Lipid utilization was reduced in p32$^{-/-}$ MEFs.

Wild type and p32$^{-/-}$ MEFs were treated with 0.5 mM FFA for 24 hours to measure the accumulation of lipid droplets. The cell culture media were changed to normal media, incubated for the indicated amount of times (0 and 24 hours) stained with Nile red and subjected to FACS analysis to quantify the amount of lipids. Average fluorescence from 4 independent experiments were presented with S.D. as error bars. Student's t-test, **p<0.01, ***p<0.001, n.s. = non-significant.

Supplementary Figure S9. Complex II inhibition did not change mitochondrial ROS production and cell viability in p32$^{-/-}$ MEFs.

(a) The histogram of flow cytometry analysis data of mitochondrial ROS obtained using MitoSox. Wild type and p32$^{-/-}$ MEFs were treated with/without 20 μM malonic acid

(mitochondrial complex II inhibitor) for 24 h, stained with MitoSox and analyzed by FACS. **(b)** Wild type and $p32^{-/-}$ MEFs were treated with 20 μM malonic acid for the indicated time and subjected to MTT assay. The % cell viability from 3 independent experiments are graphed with S.D. as error bars. Student's t-test, ***p<0.001, n.s. = non-significant.

Supplementary Figure S10. The metabolic shift from OXPHOS to glycolysis in p32-/- MEFs.

(**a**) Wild type, $p32^{-/-}$ and $p32^{-/-}$+p32 MEFs were seeded in an XF-24 extracellular flux analyzer, and the real-time OCR were determined through sequential treatments with oligomycin, FCCP and antimycin-A/rotenone (ETC inhibitors) for Agilent Seahorse XF Cell Mito Stress Test. **(b)** Introduction of calculation methods from Agilent Seahorse XF Cell Mito Stress Test (https://www.agilent.com/cs/library/usermanuals/public/XF_Cell_Mito_Stress_Test_Kit_User_Guide.pdf). **(c)** Wild type, $p32^{-/-}$ and $p32^{-/-}$+p32 MEFs MEFs were seeded in an XF-24 extracellular flux analyzer, and the real-time ECAR was determined during sequential treatments with Glucose, oligomycin and 2-DG (glycolysis inhibitor) for Agilent Seahorse XF Glycolysis Stress Test. **(d)** Introduction of calculation methods from Agilent Seahorse XF Glycolysis Stress Test (https://www.agilent.com/cs/library/usermanuals/public/XF_Glycolysis_Stress_Test_Kit_User_Guide.pdf).

Supplementary Figure S11. p32/C1QBP deficiency induced apoptosis in HCT116 cells.

The representative FACS plots of Figure 7c. HCT116-pLKO and two independent HCT116-sh-p32 cells were stained with Annexin-V/PI and analyzed by FACS.

Supplementary Figure S12. p32/C1QBP deficiency did not induce apoptosis in monolayer cultured MEFs.

Wild type and $p32^{-/-}$ MEFs were subjected to immunoblotting assay for indicated proteins.

Supplementary Figure S13. p32/C1QBP deficiency did not induce HIF-1α.

(a) Monolayer cultured Wild type and $p32^{-/-}$ MEFs were subjected to Western blotting for indicated proteins. **(b)** Spheroids of wild type and $p32^{-/-}$ MEFs were subjected to Western blotting for indicated proteins.

References

1 McClatchey, P. M., Keller, A. C., Bouchard, R., Knaub, L. A. & Reusch, J. E. Fully automated software for quantitative measurements of mitochondrial morphology. *Mitochondrion* **26**, 58-71, doi:10.1016/j.mito.2015.12.003 (2016).

2 Otsu, N. A threshold selection method from the gray-level histogram. *IEEE Transactions on Systems, Man, and Cybernetics* **9**, 62-66 (1979).

Supplementary Figure 1

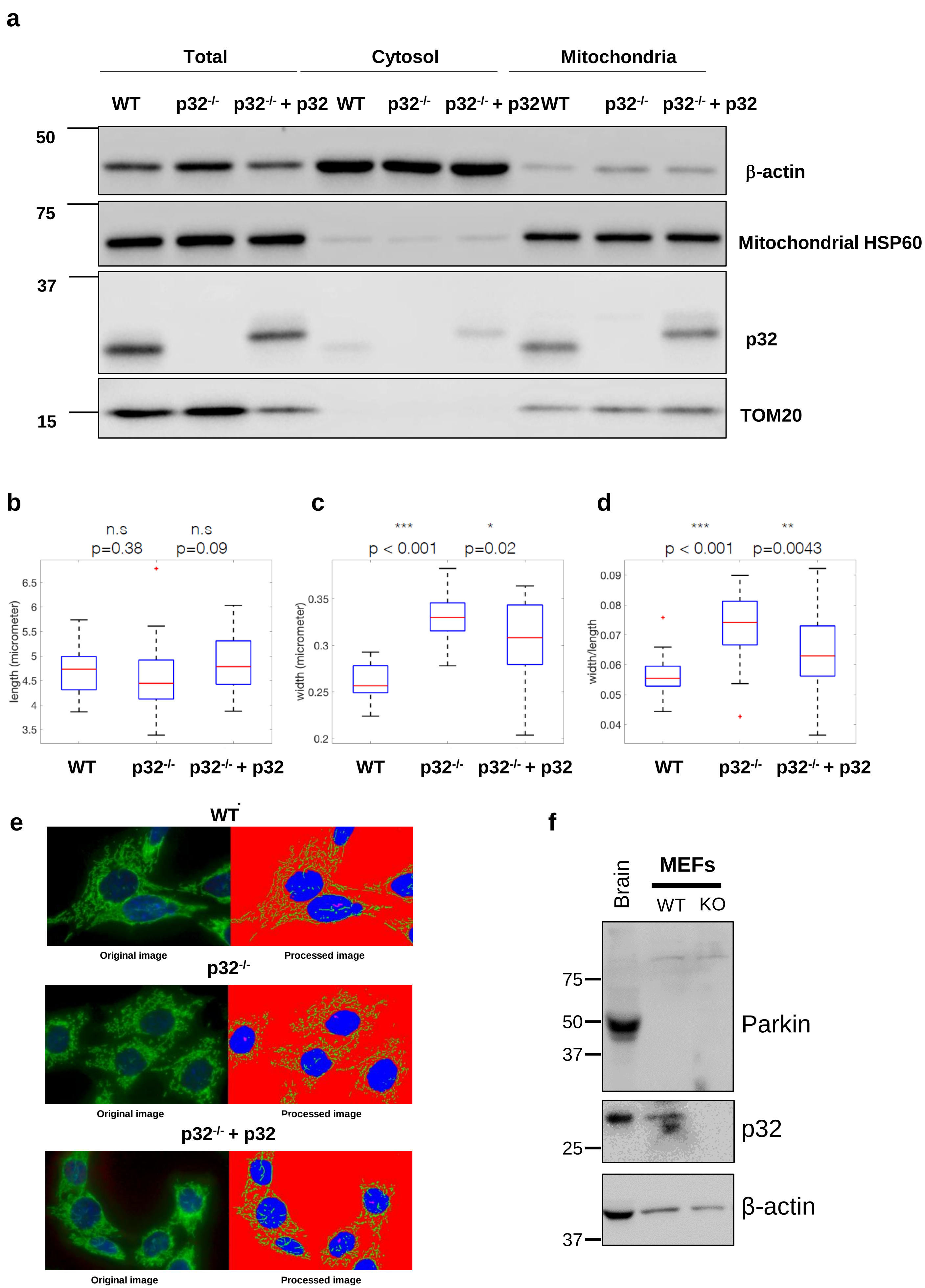

Supplementary Figure 2

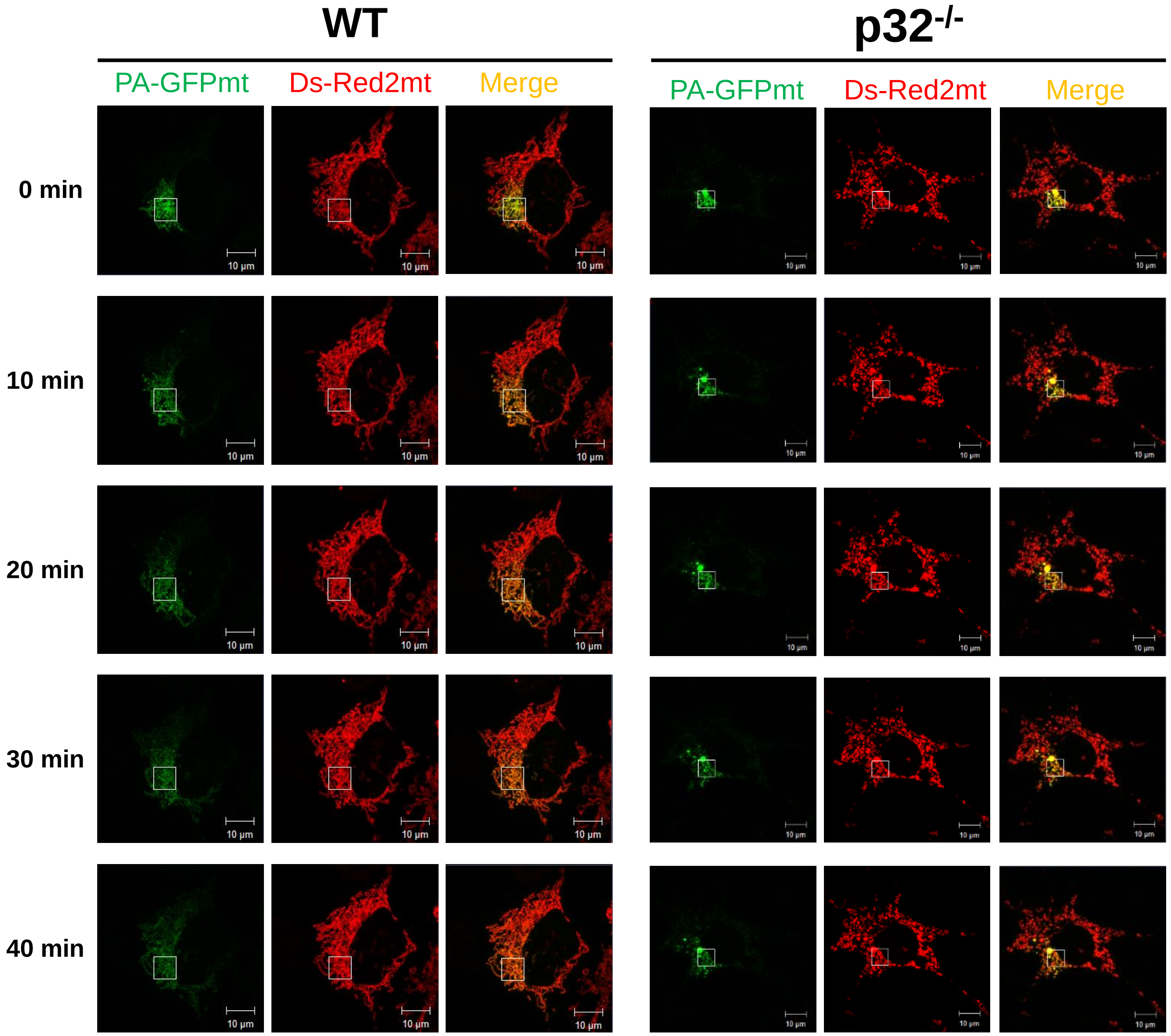

Supplementary Figure 3

a

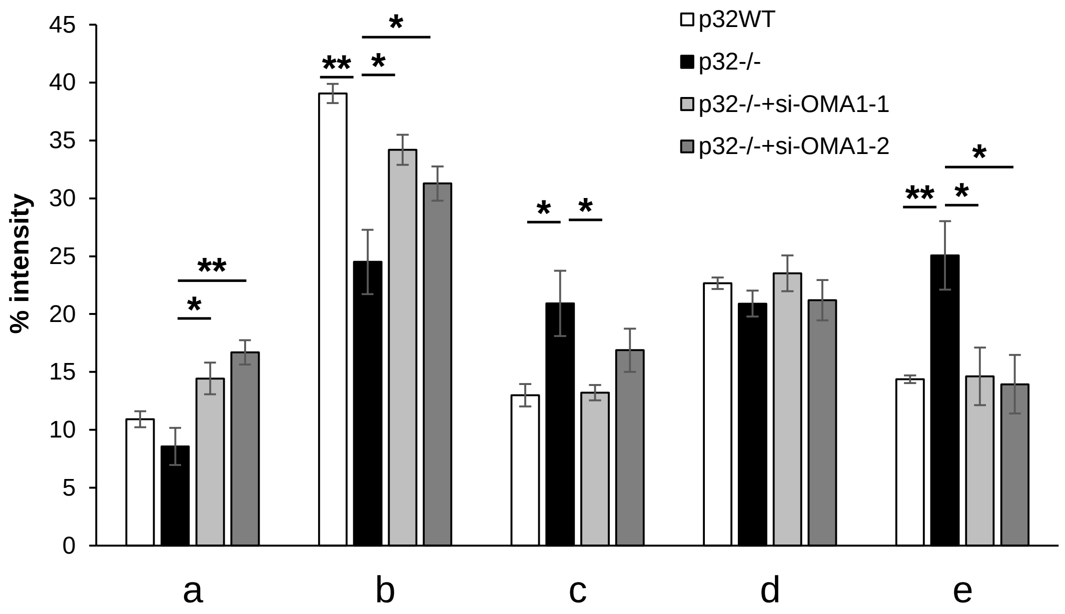

b

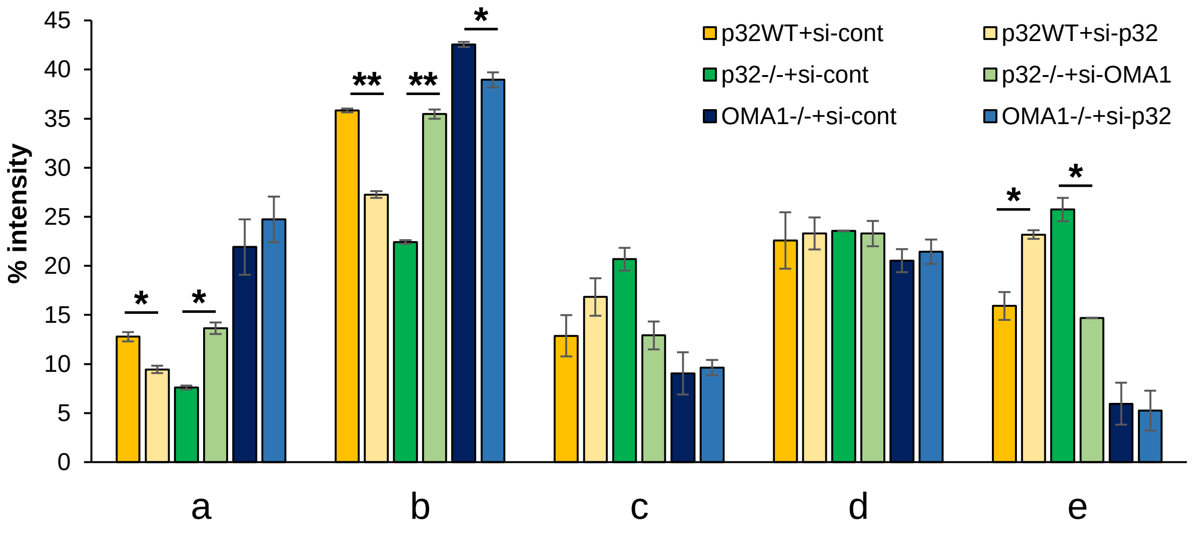

Supplementary Figure 4

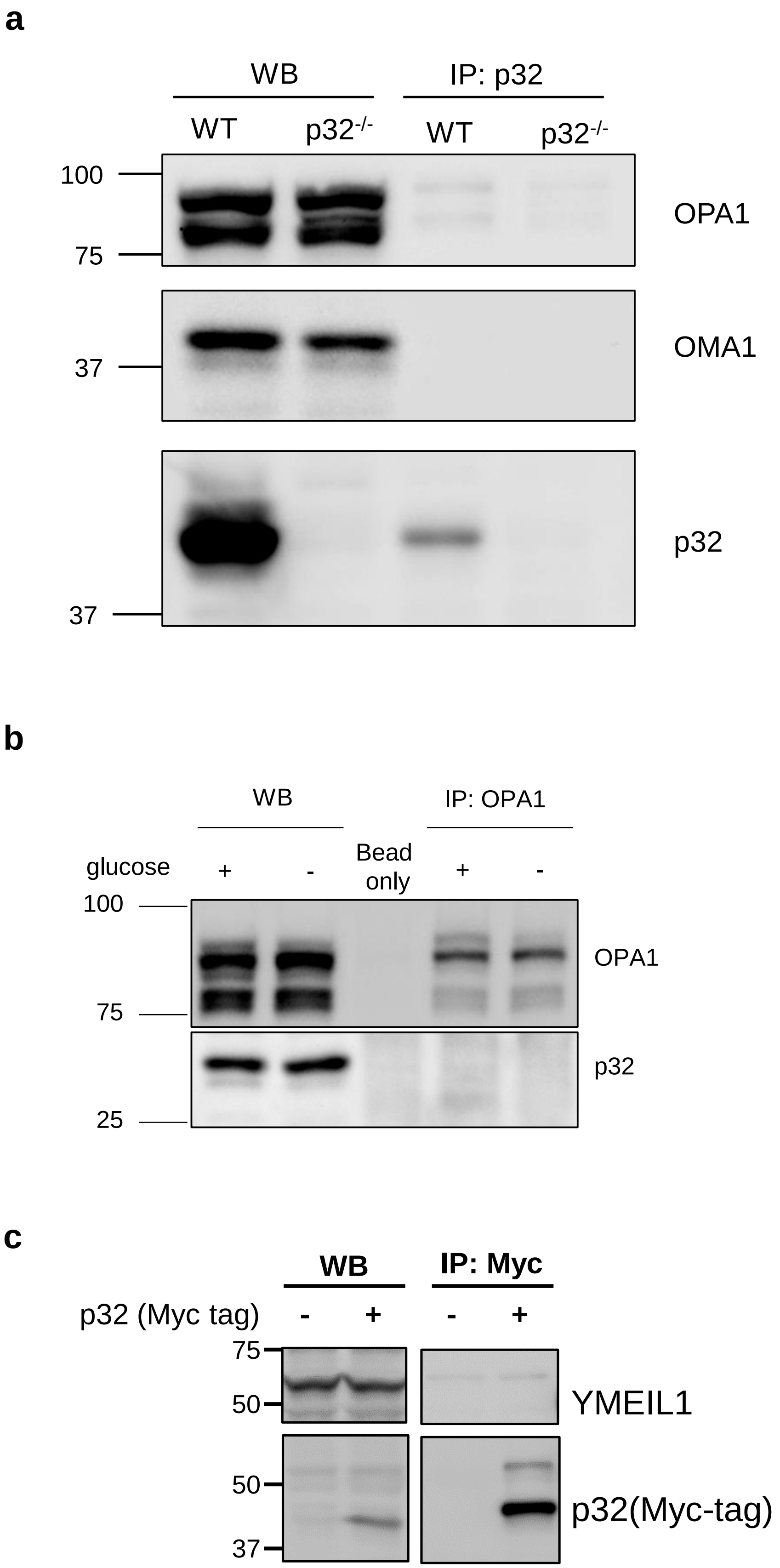

Supplementary Figure 5

a

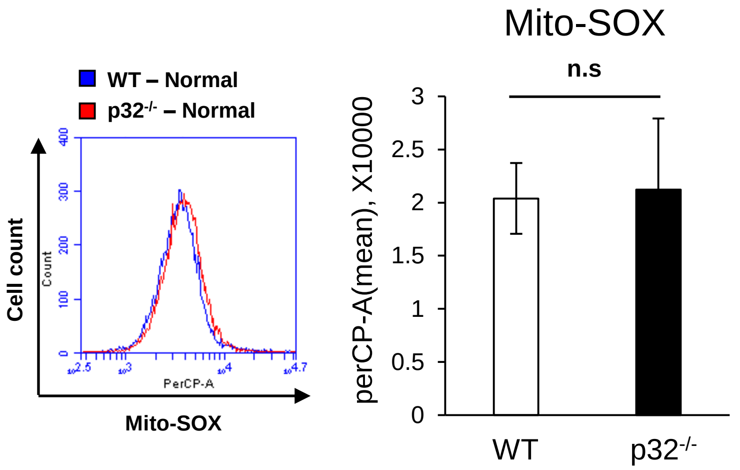

b

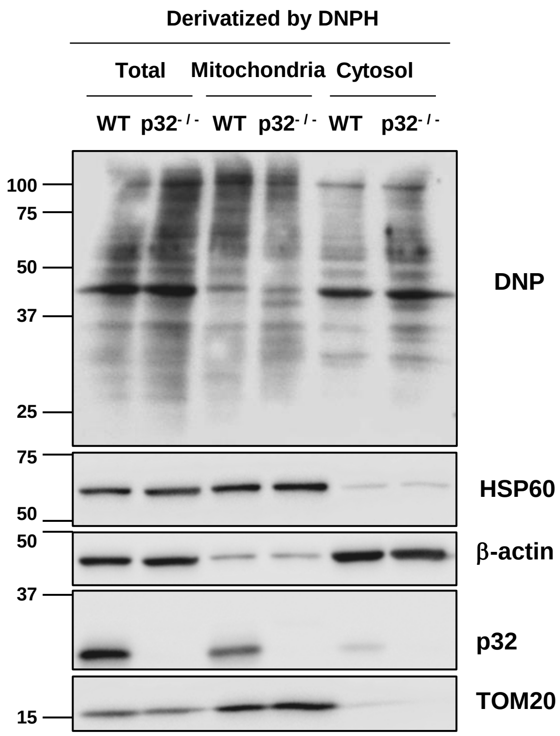

c

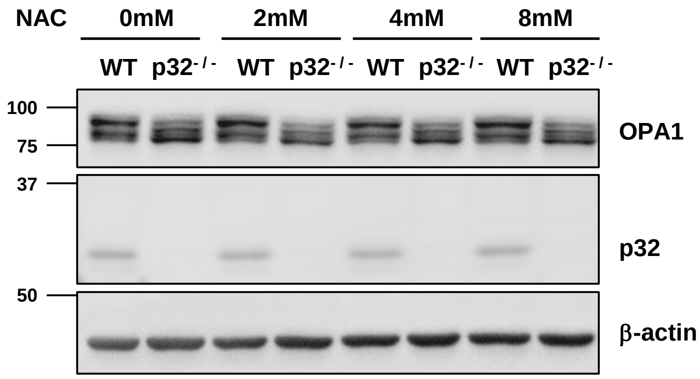

d

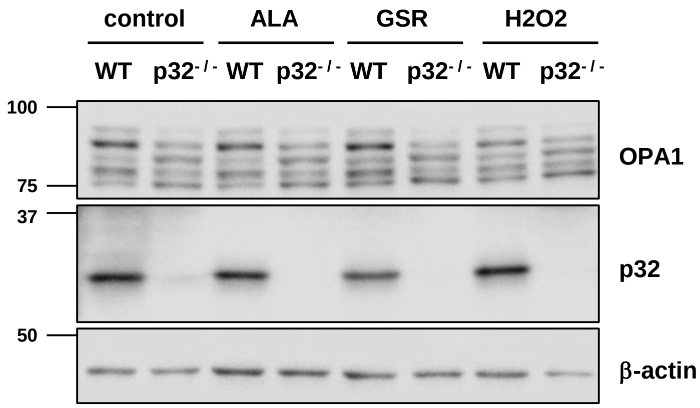

Supplementary Figure 6

a

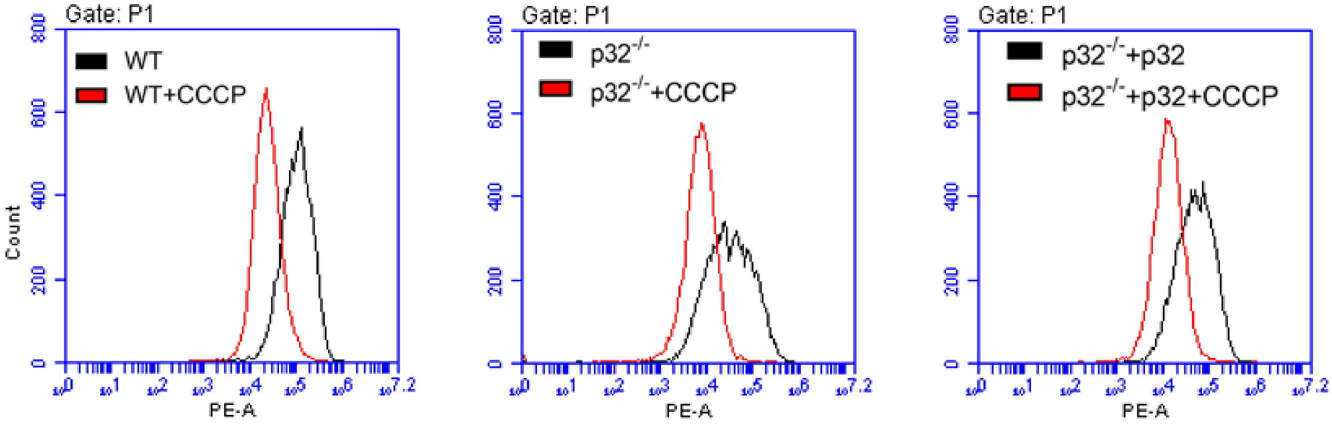

b

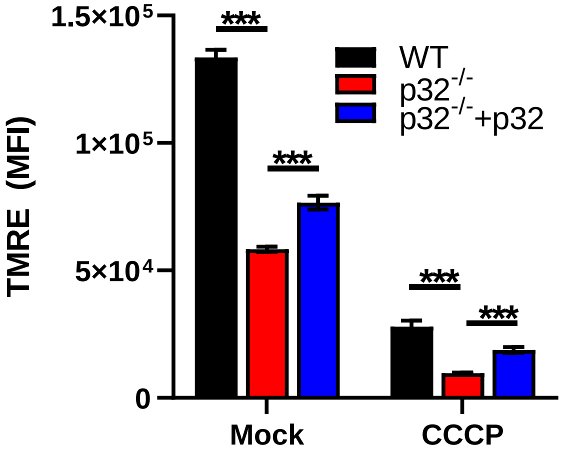

Supplementary Figure 7

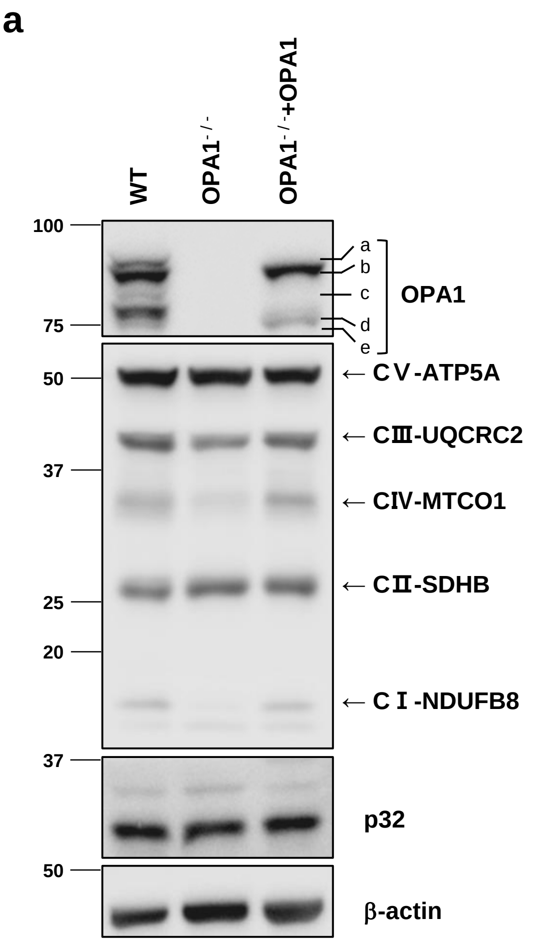

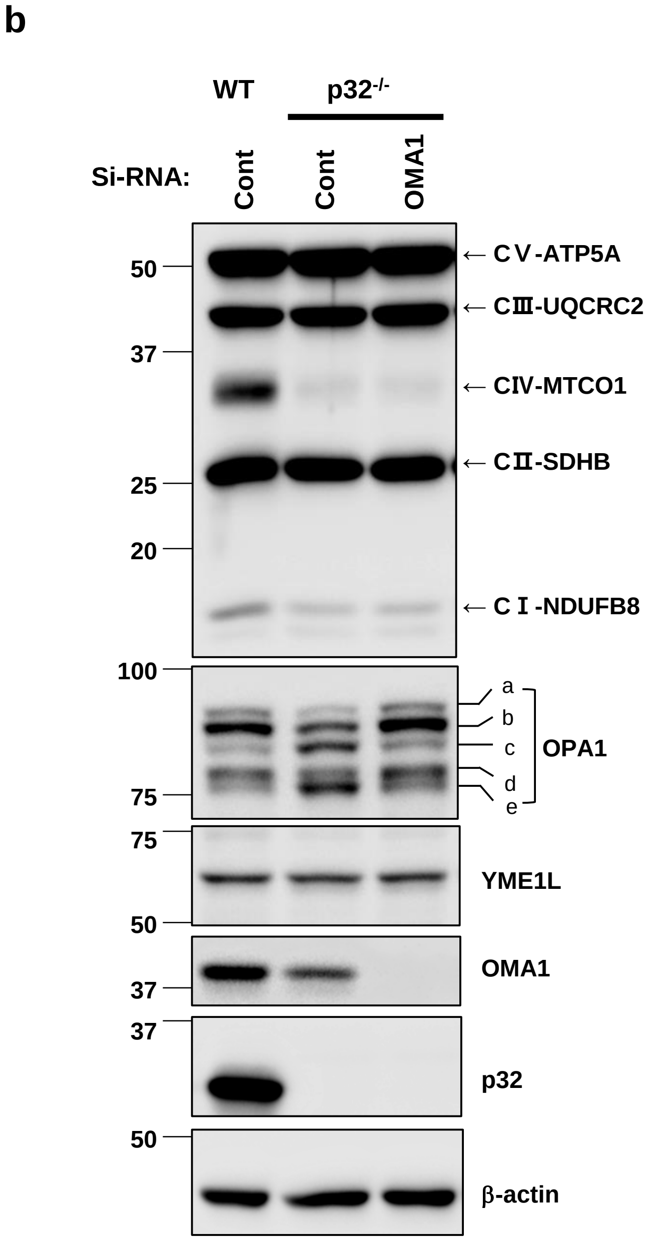

Supplementary Figure 8

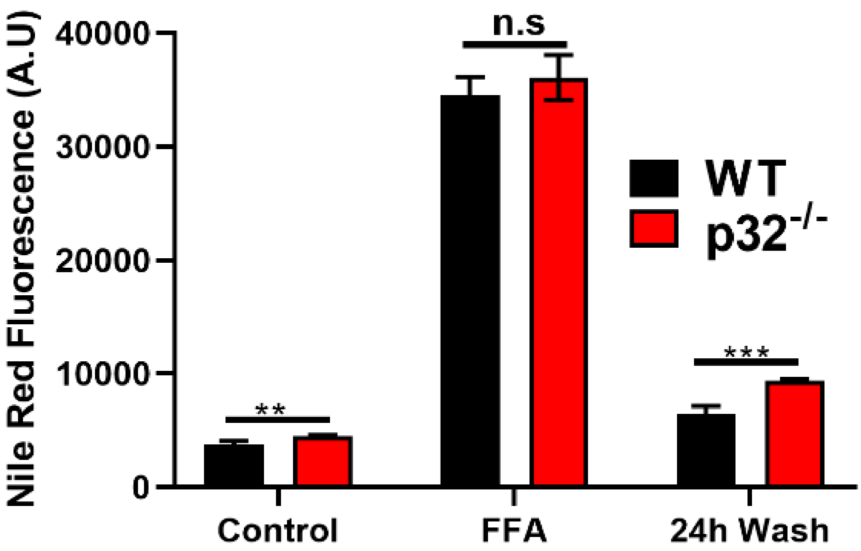

Supplementary Figure 9

a

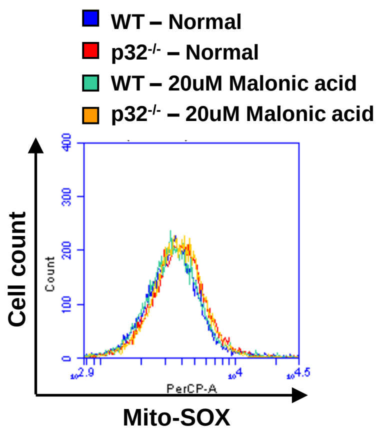

b

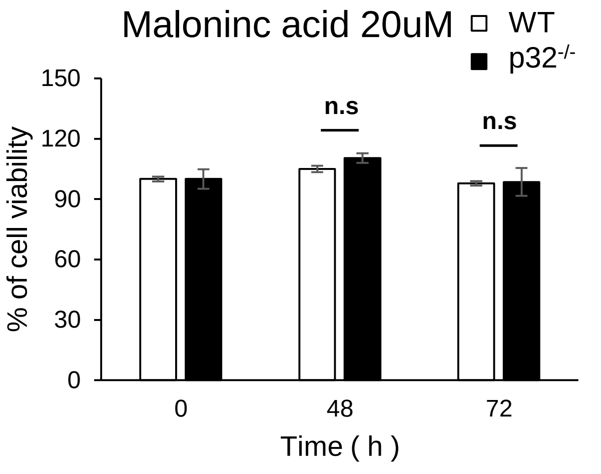

Supplementary Figure 10

a

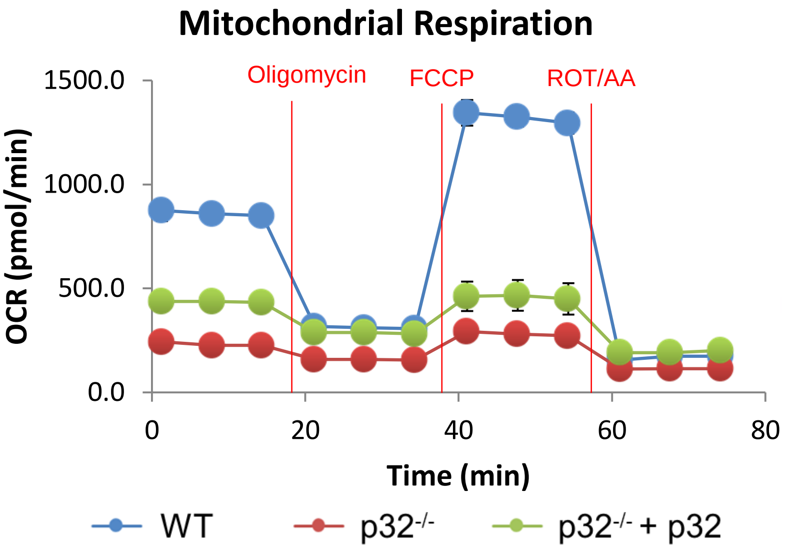

b

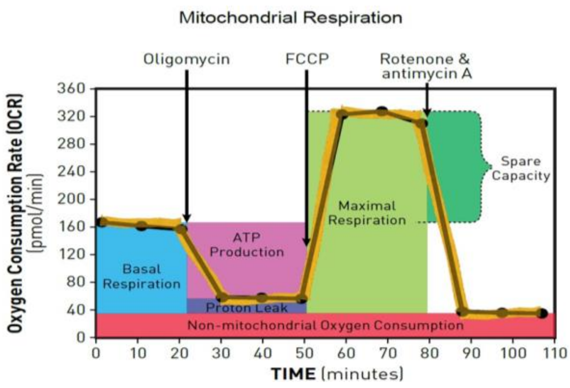

c

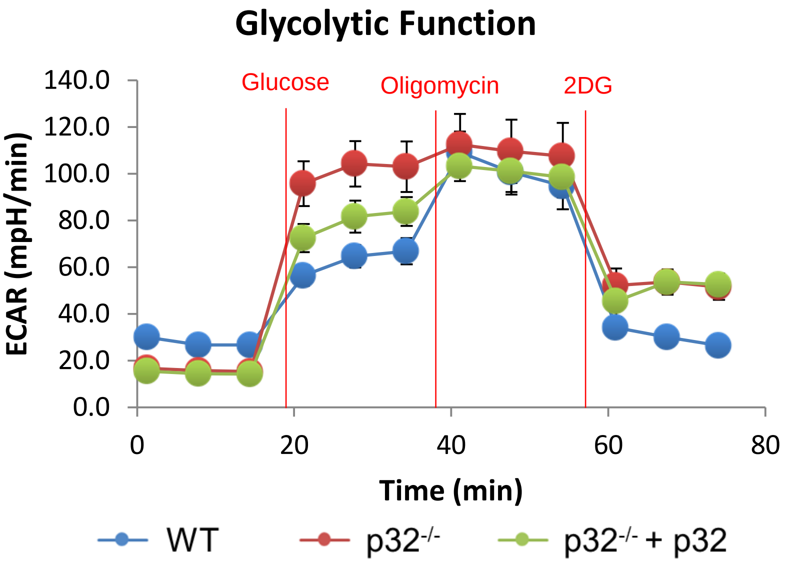

d

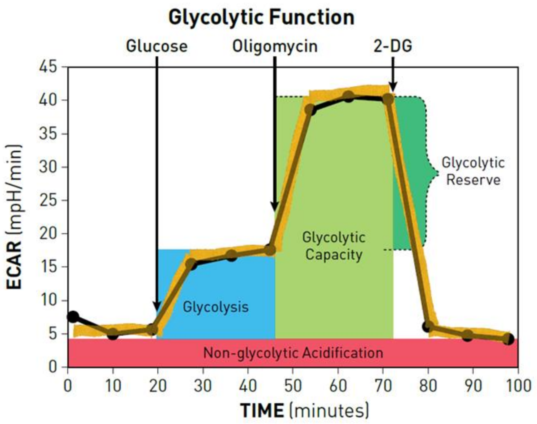

Supplementary Figure 11

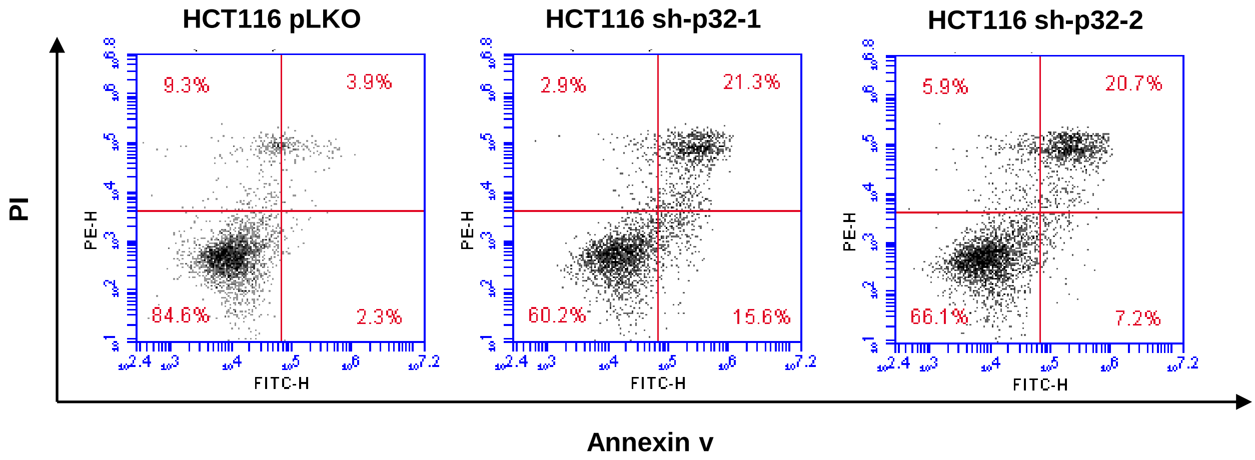

Supplementary Figure 12

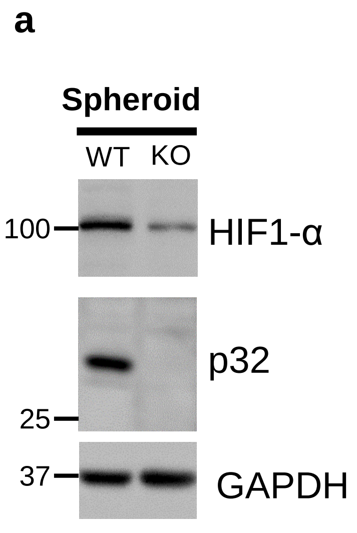

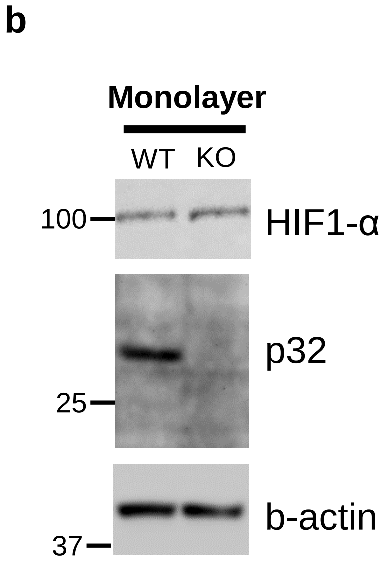

Supplementary Figure 13

a

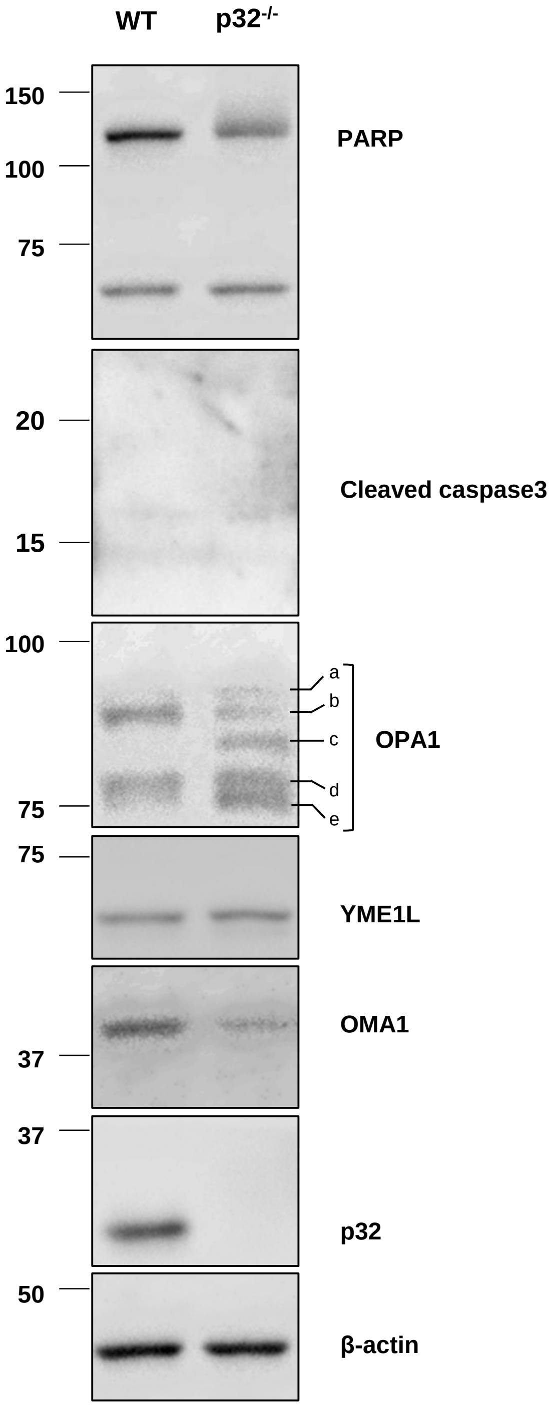

Whole blot for cropped image

Whole blot for cropped image for figure 3a

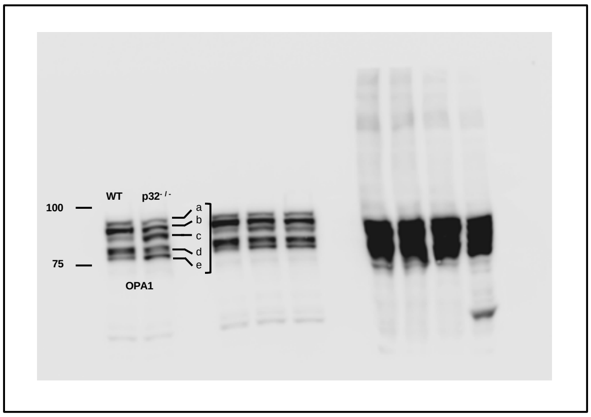

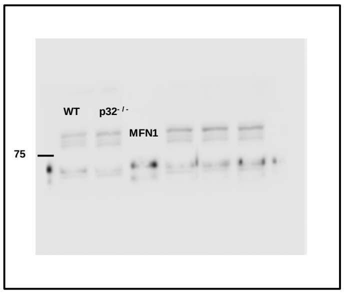

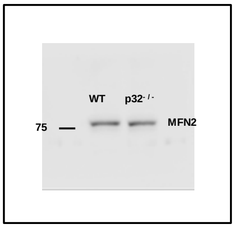

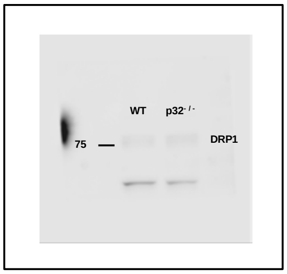

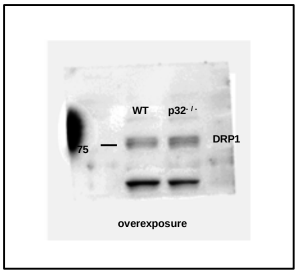

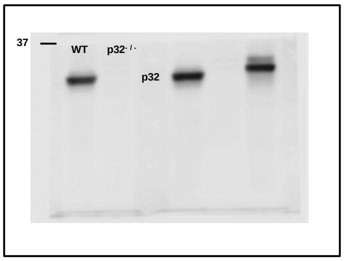

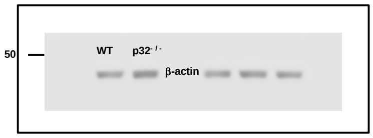

Whole blot for cropped image for figure 3b

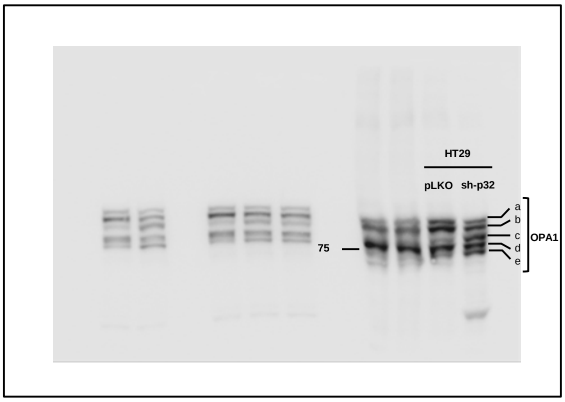

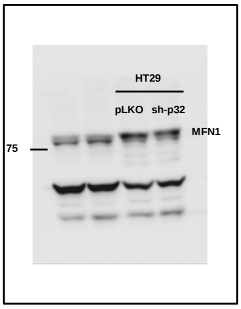

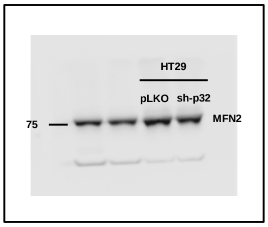

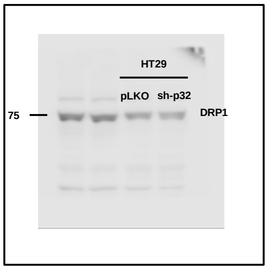

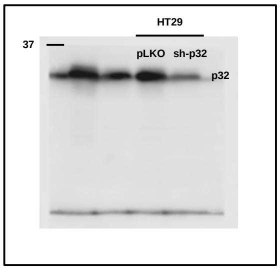

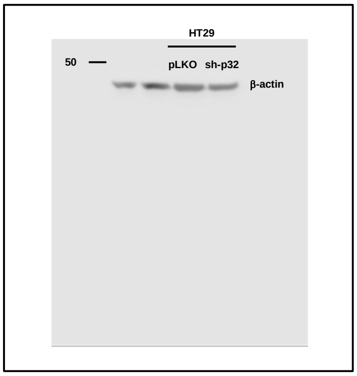

Whole blot for cropped image for figure 3c

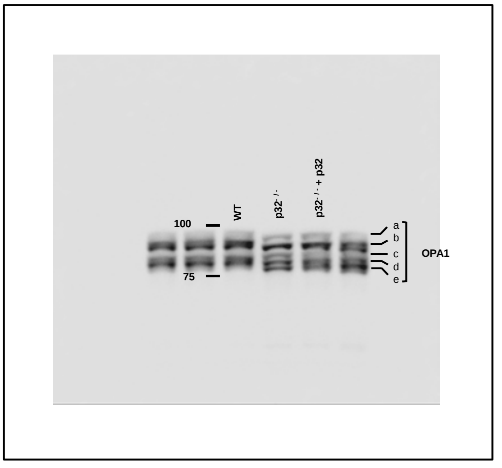

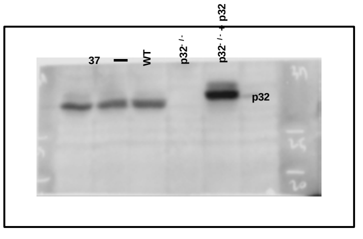

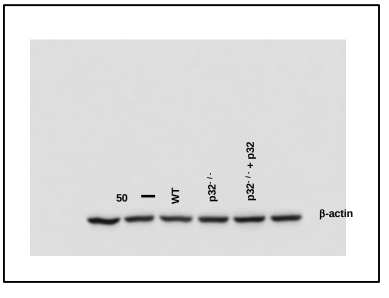

Whole blot for cropped image for figure 4a

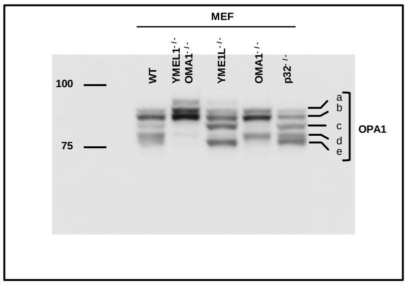

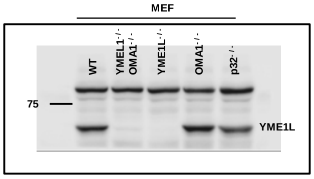

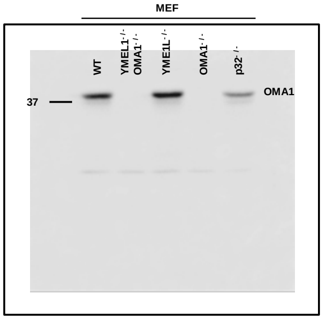

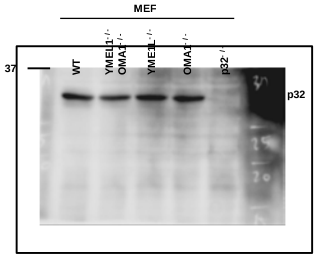

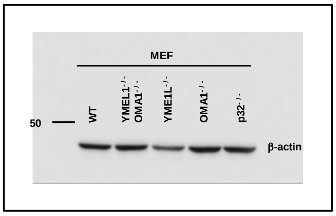

Whole blot for cropped image for figure 4b

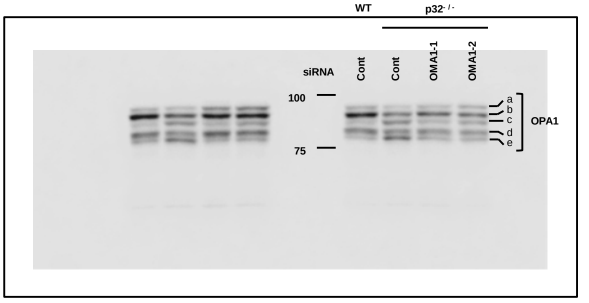

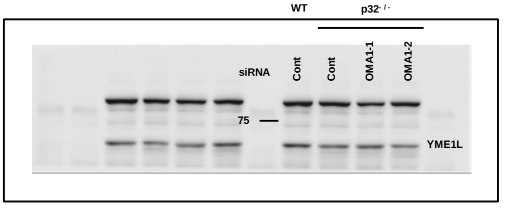

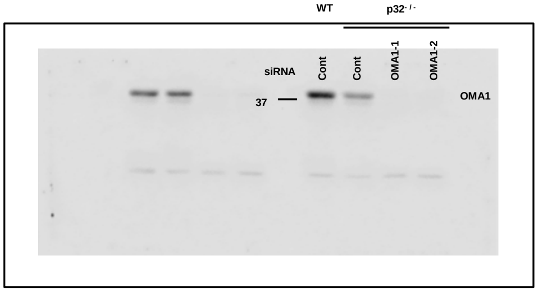

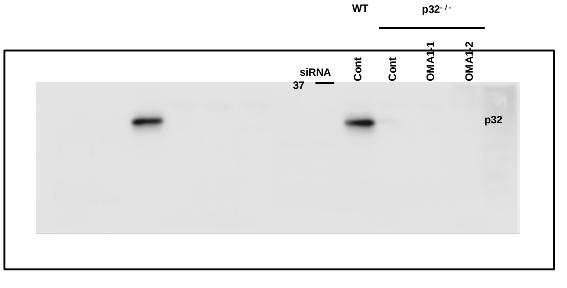

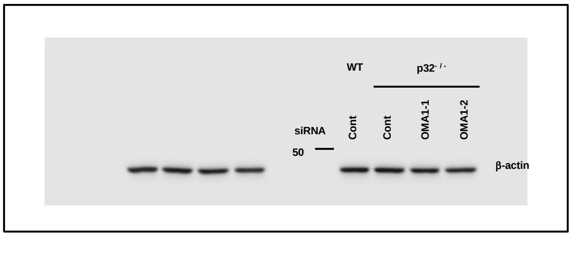

Whole blot for cropped image for figure 4c

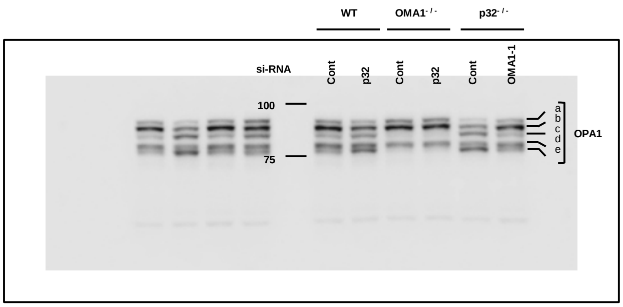

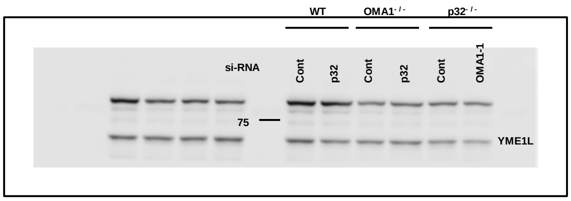

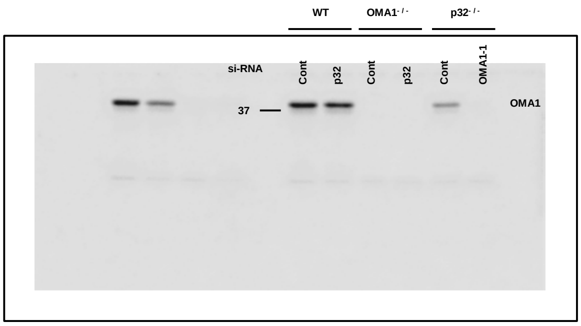

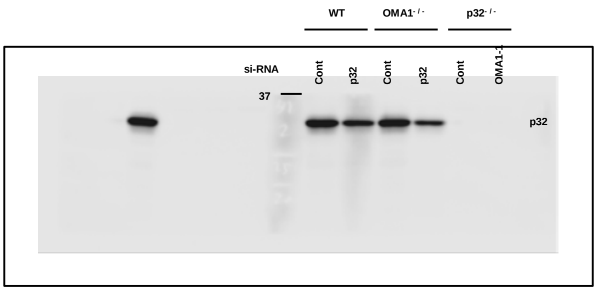

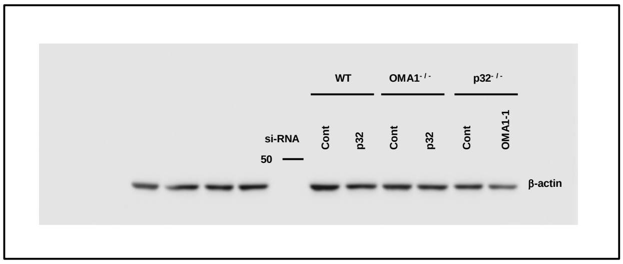

Whole blot for cropped image for figure 5b

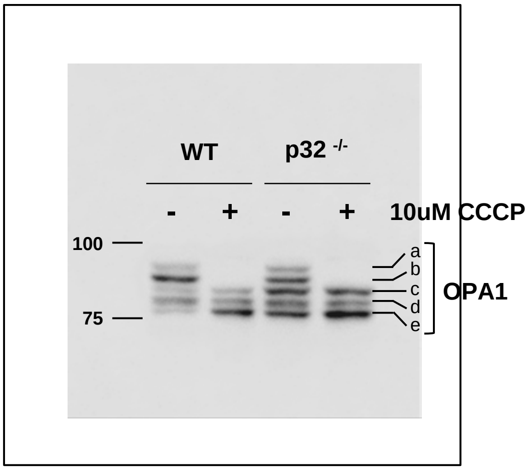

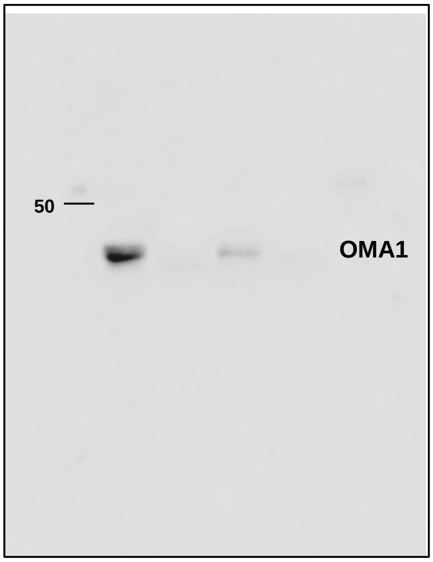

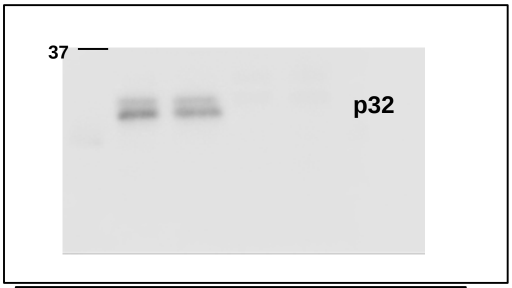

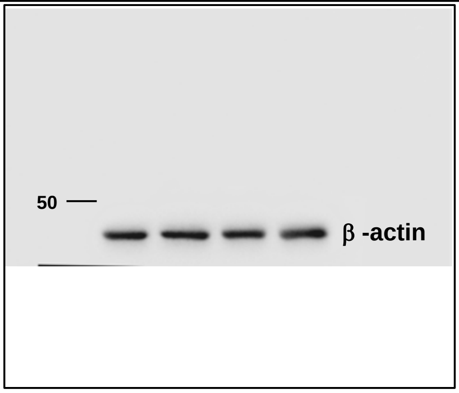

Whole blot for cropped image for figure 5c

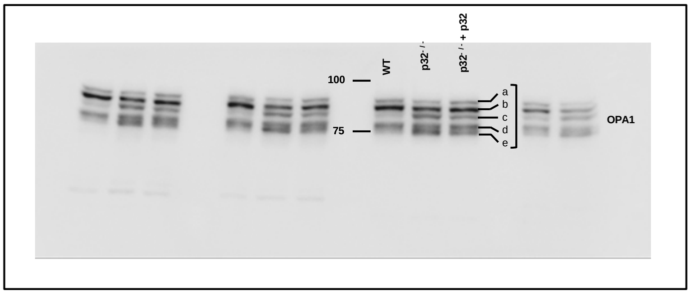

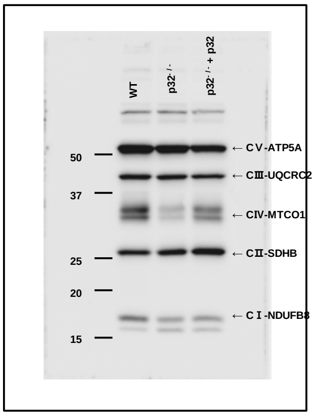

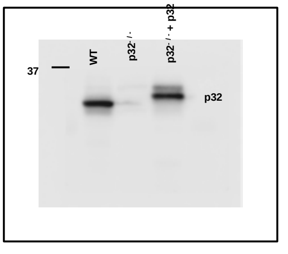

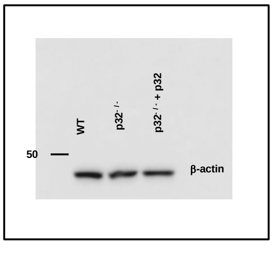

Whole blot for cropped image for figure 5e

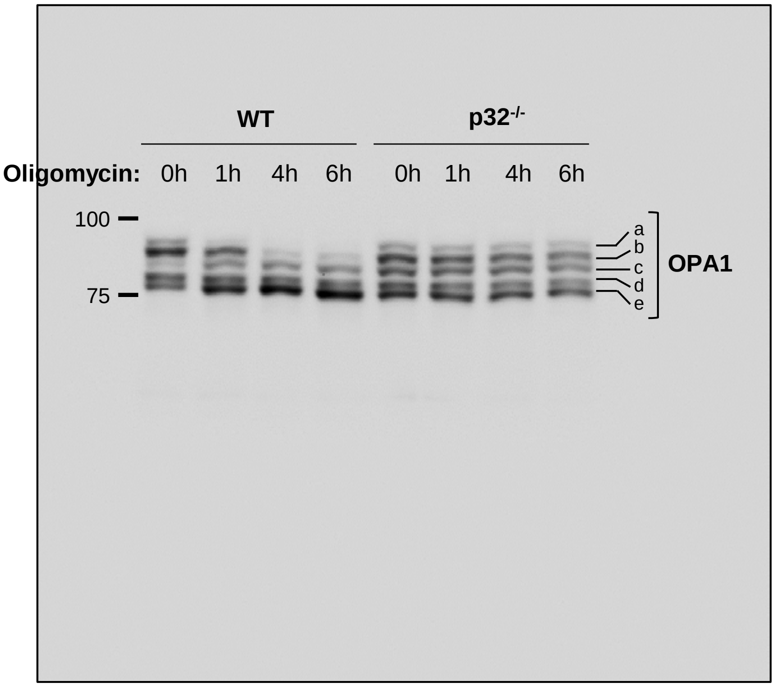

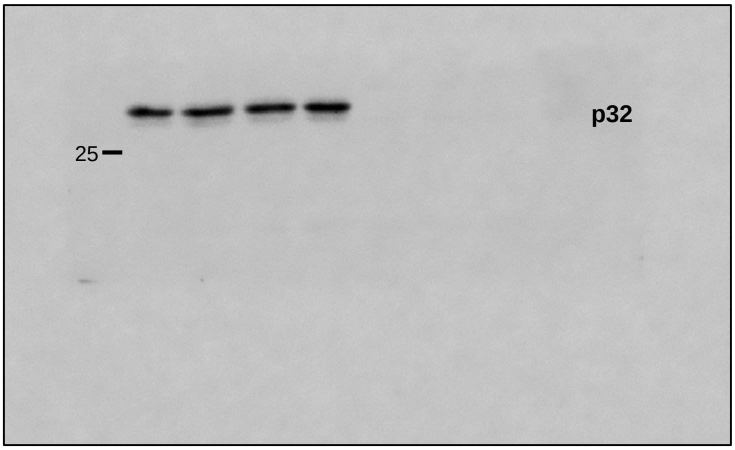

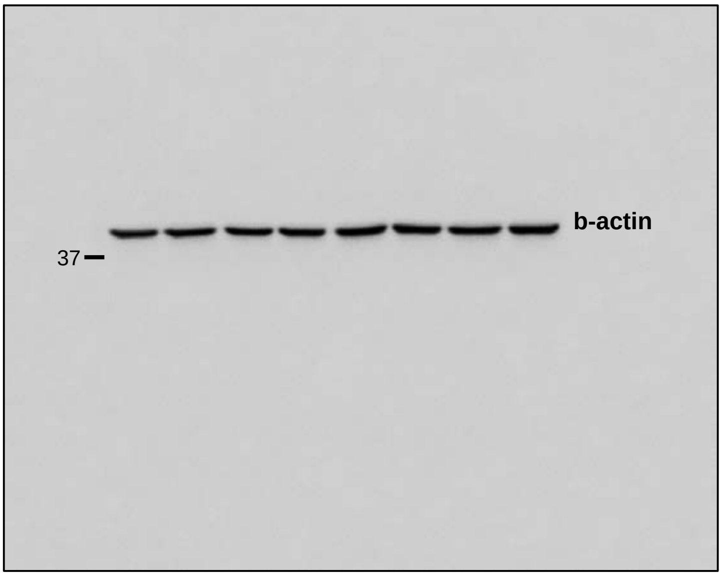

Whole blot for cropped image for figure 5f

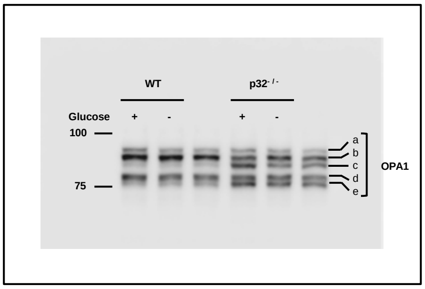

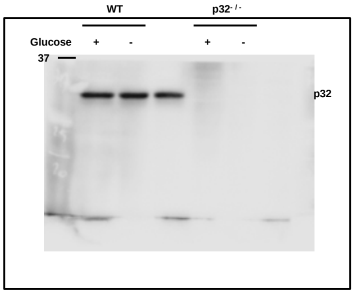

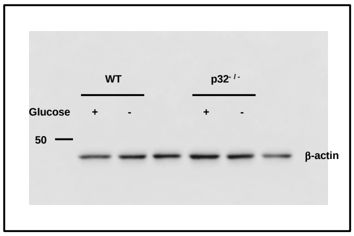

Whole blot for cropped image for figure 7c

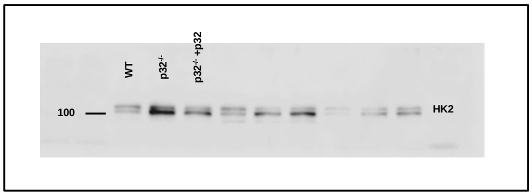

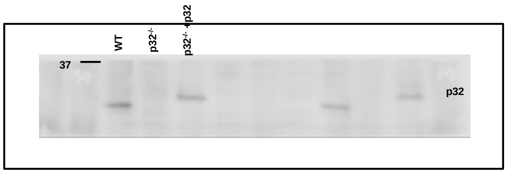

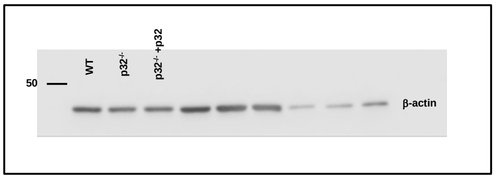

Whole blot for cropped image for figure 8d

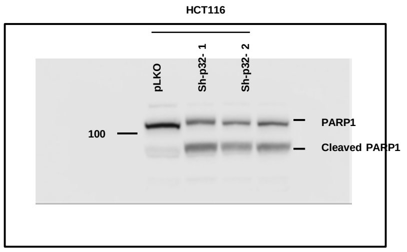

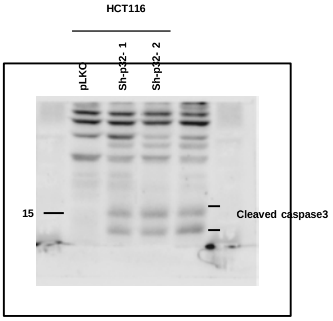

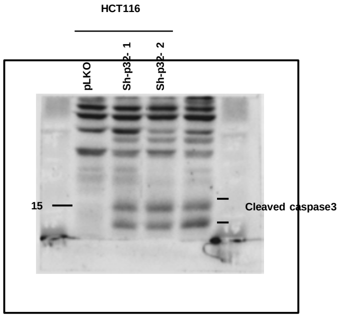

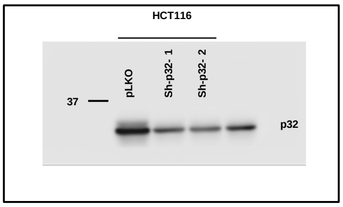

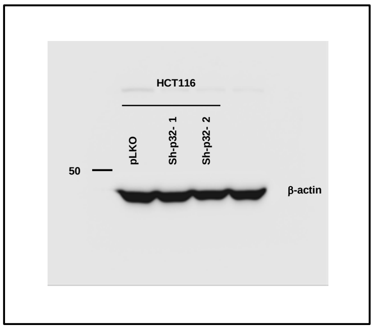

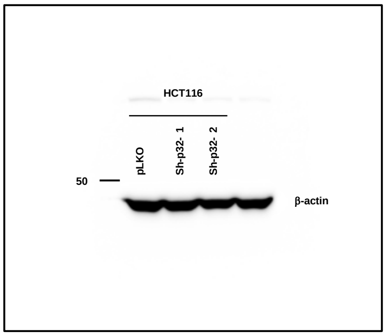

Supplementary Figure 1a

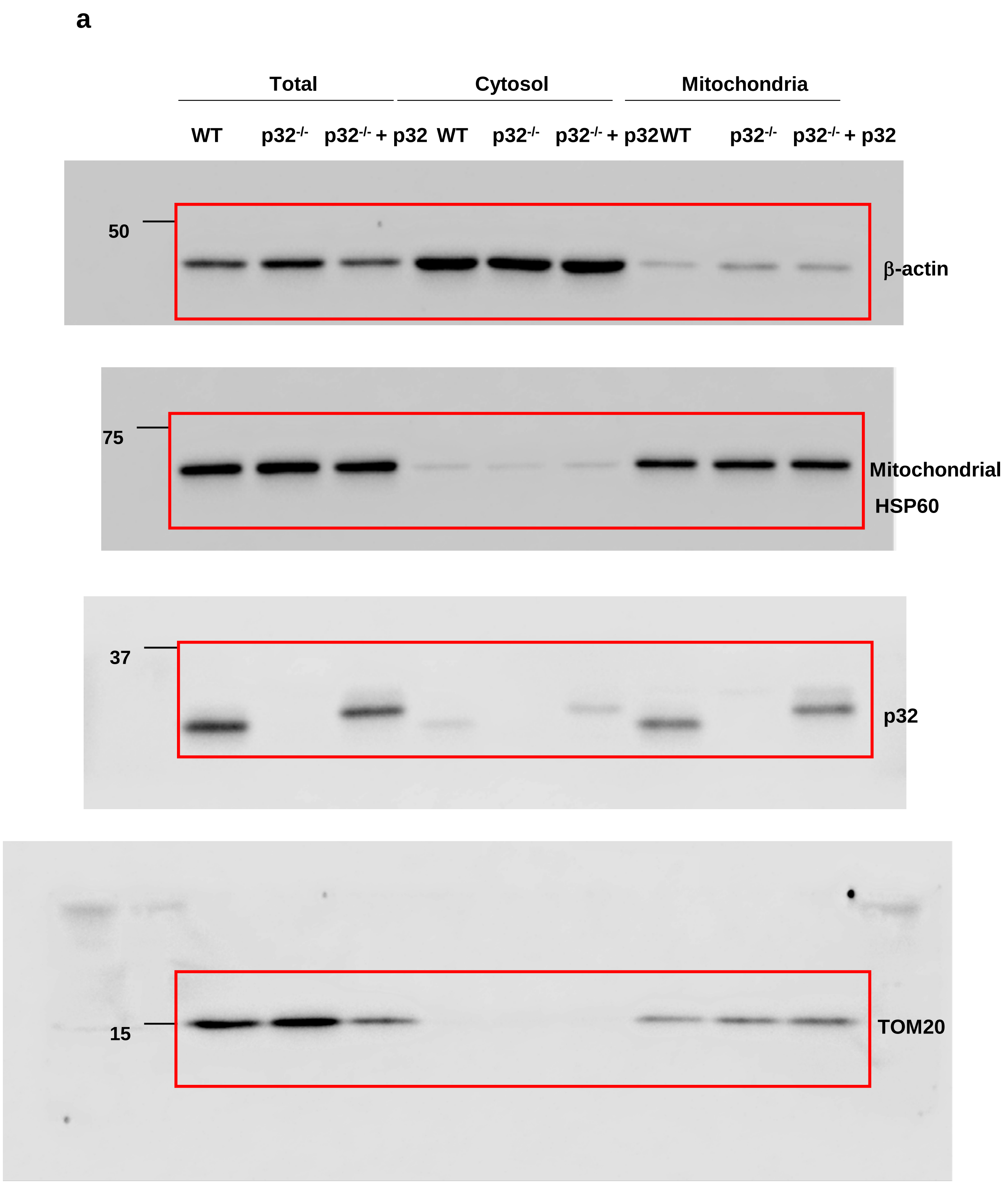

Supplementary Figure 1f

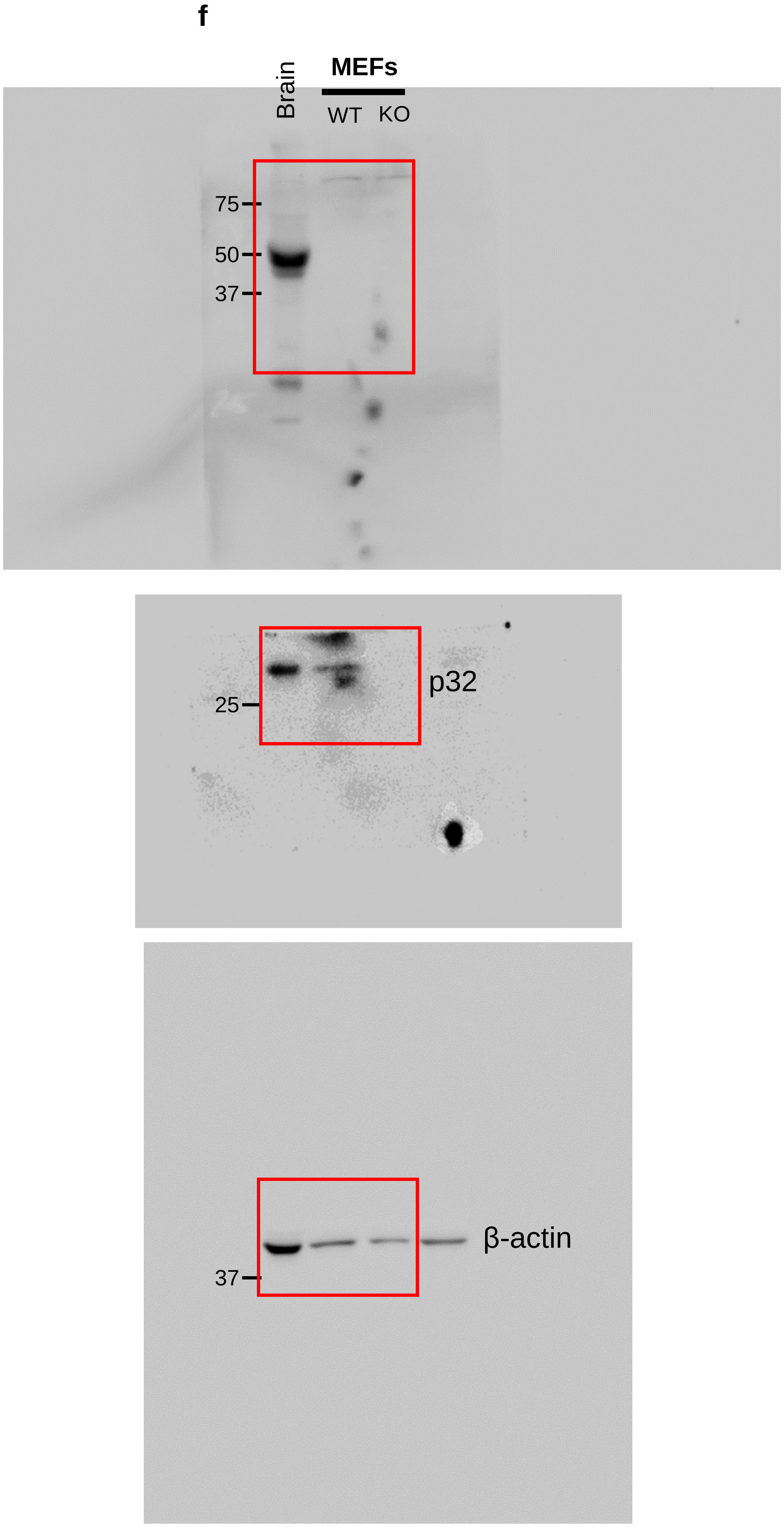

Supplementary Figure 4a

a

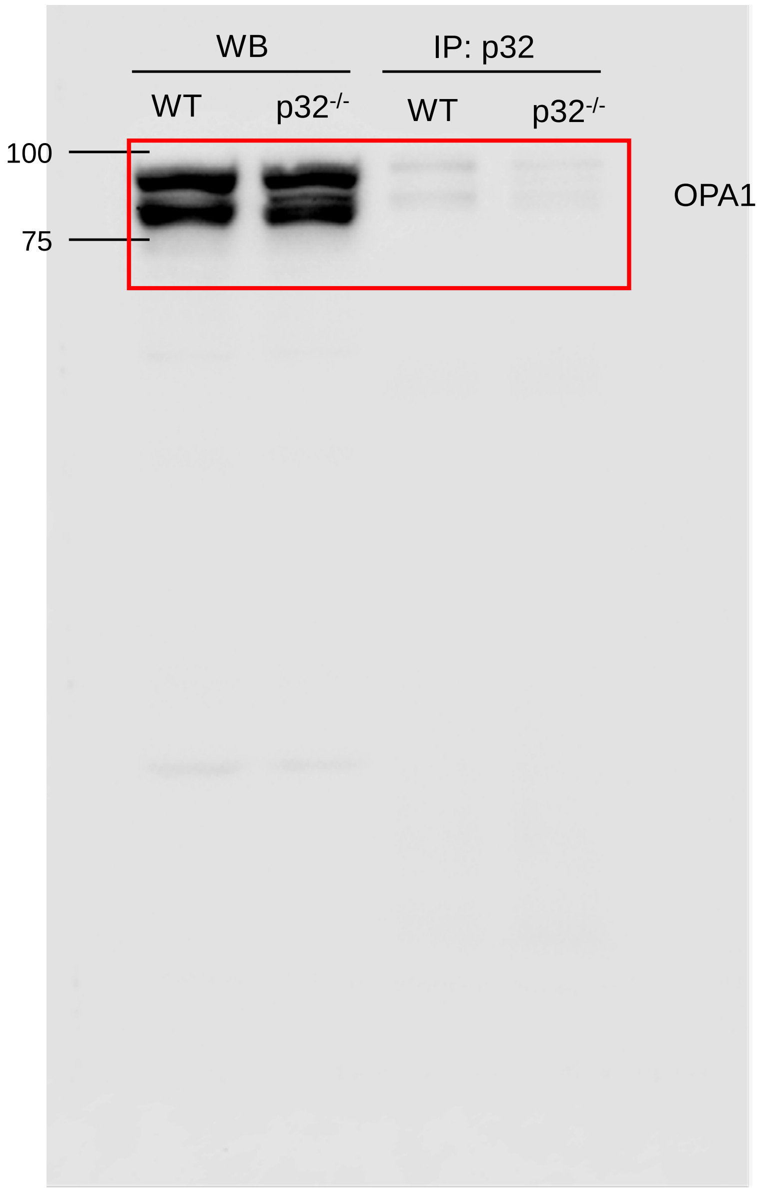

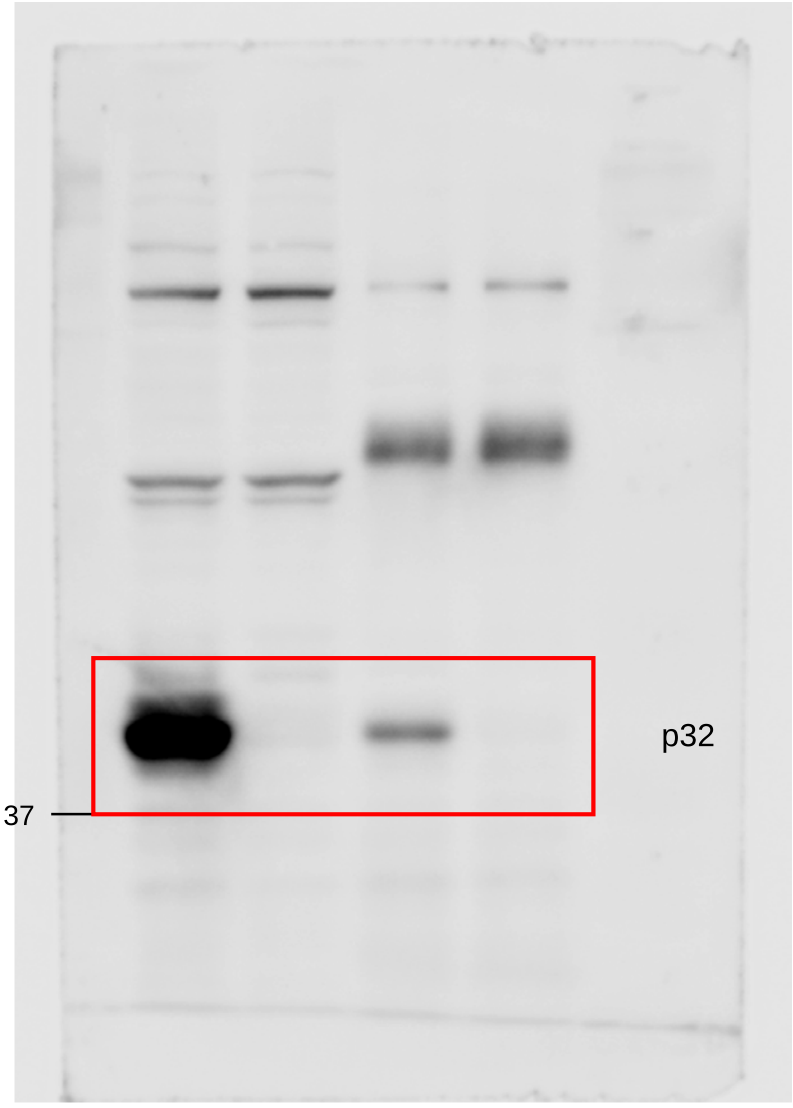

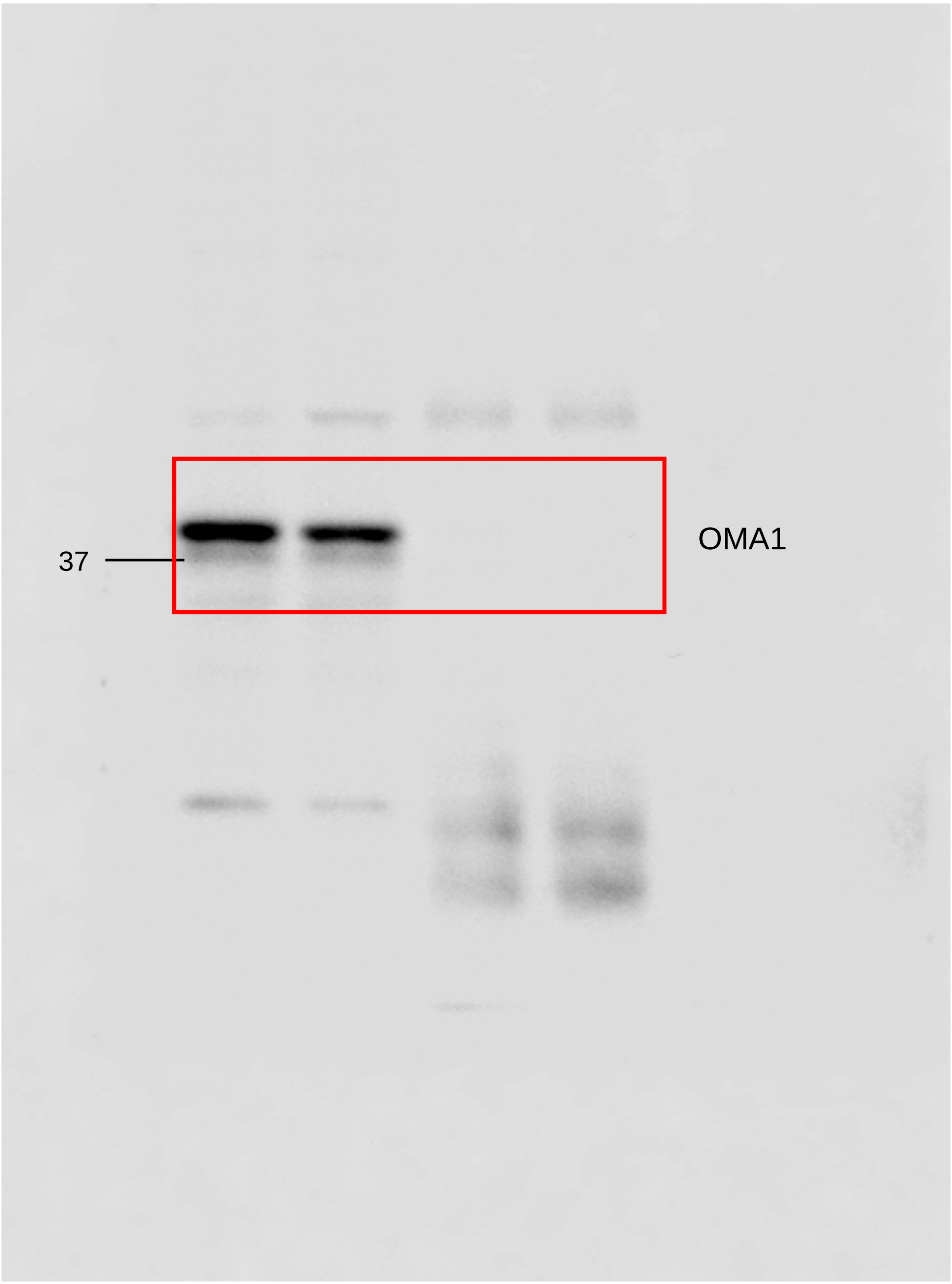

Supplementary Figure 4b

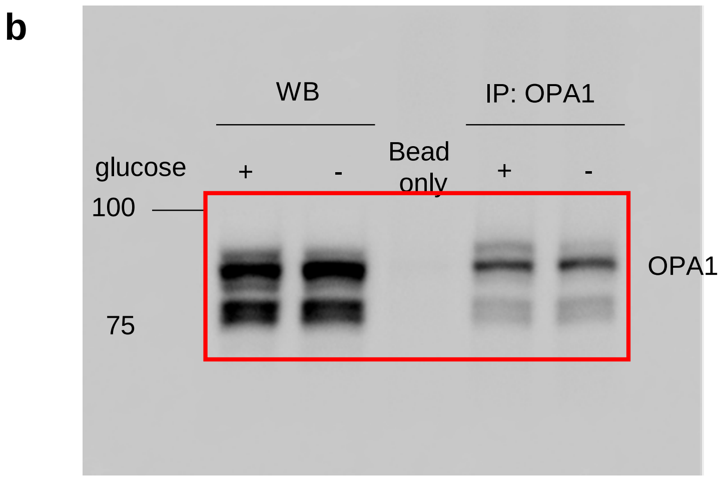

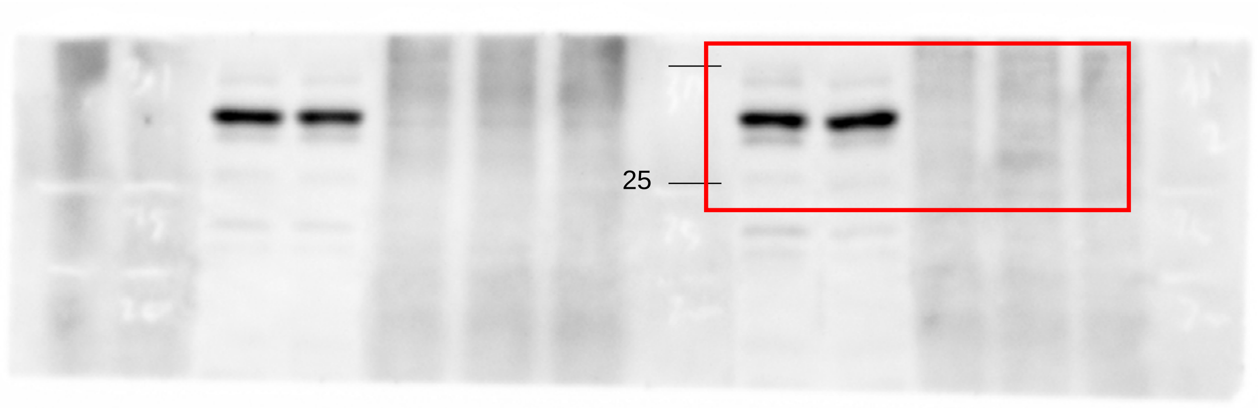

Supplementary Figure 4c

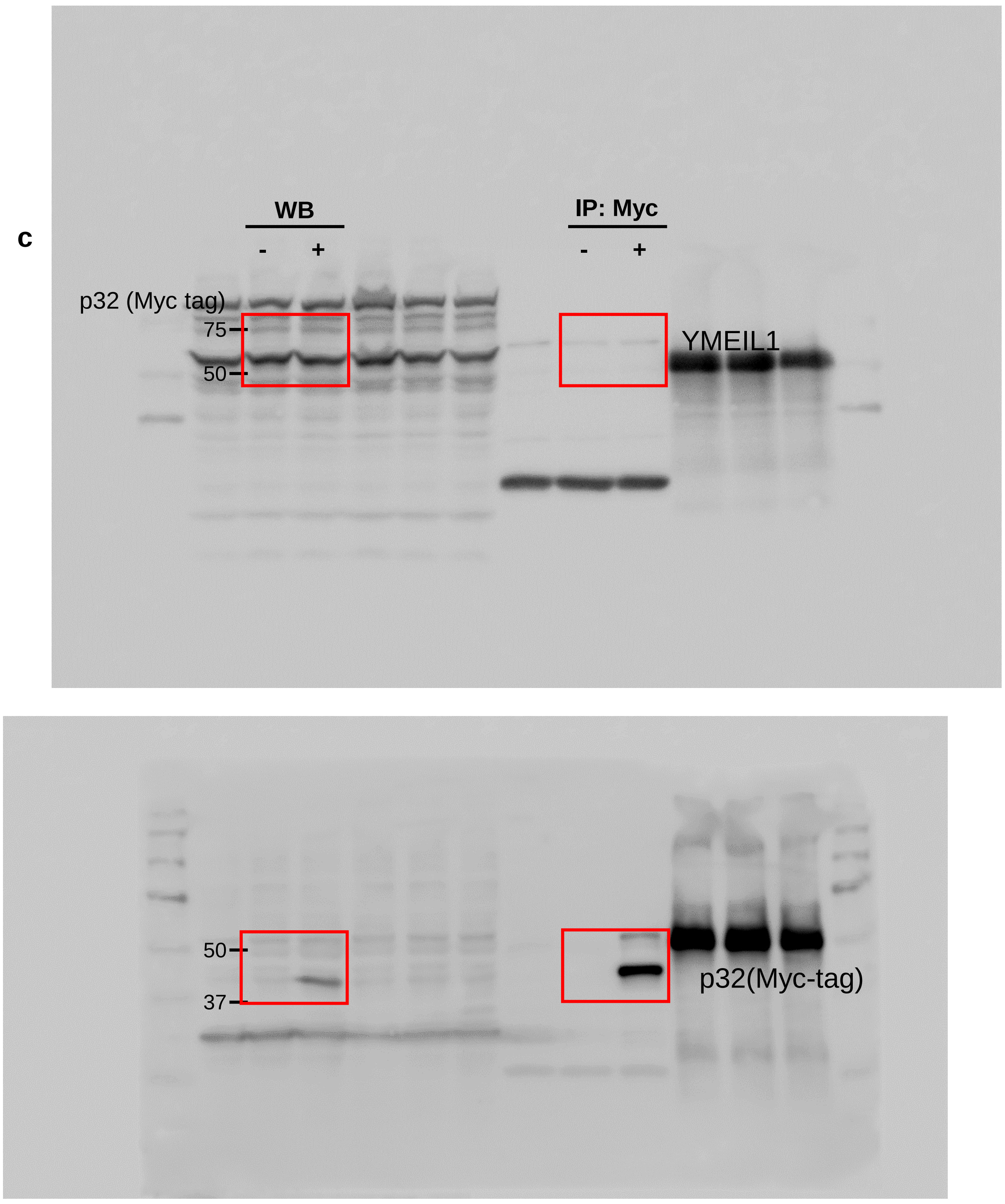

Supplementary Figure 5b

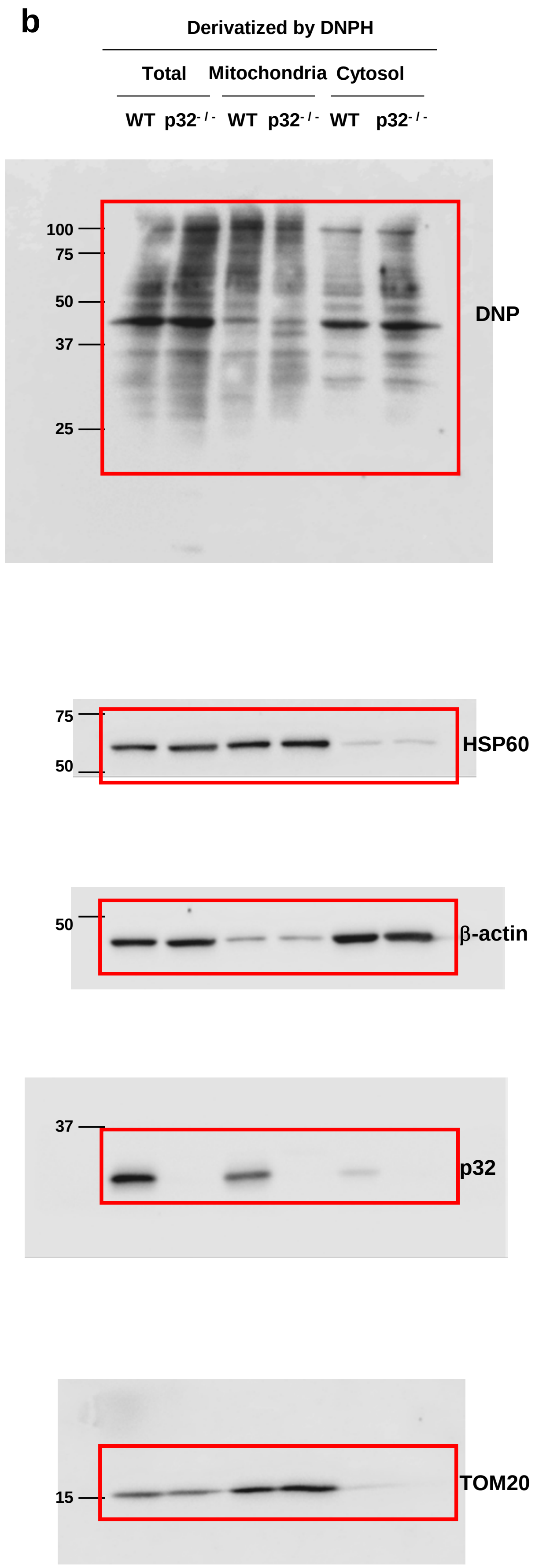

Supplementary Figure 5c

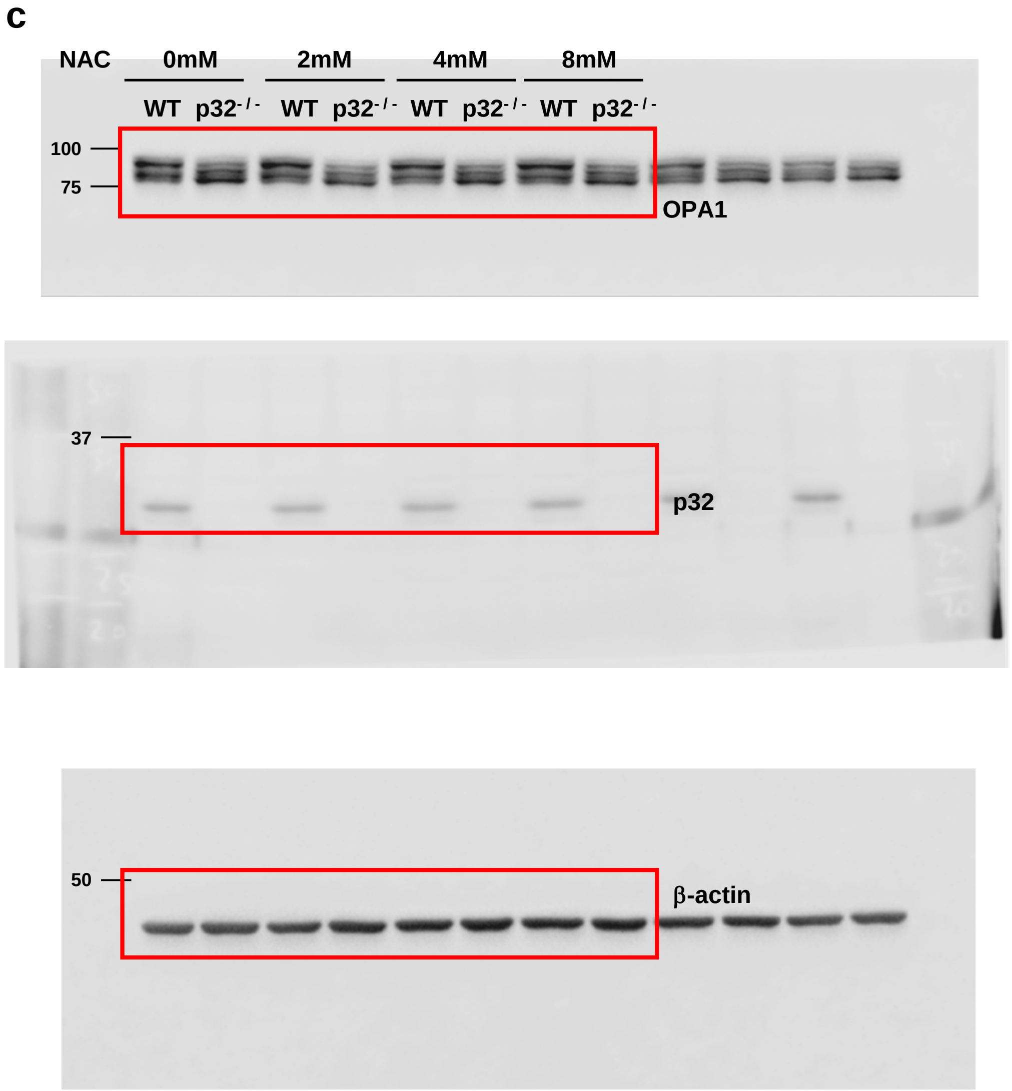

Supplementary Figure 5d

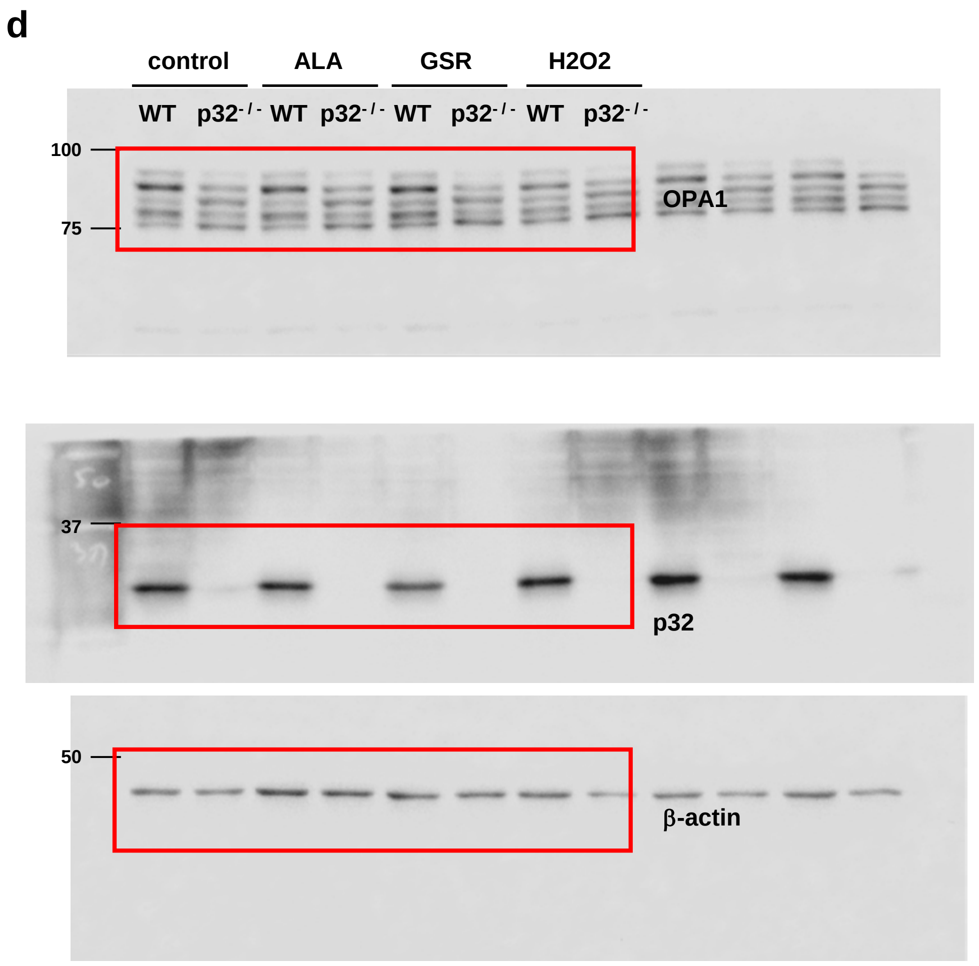

Supplementary Figure 7a

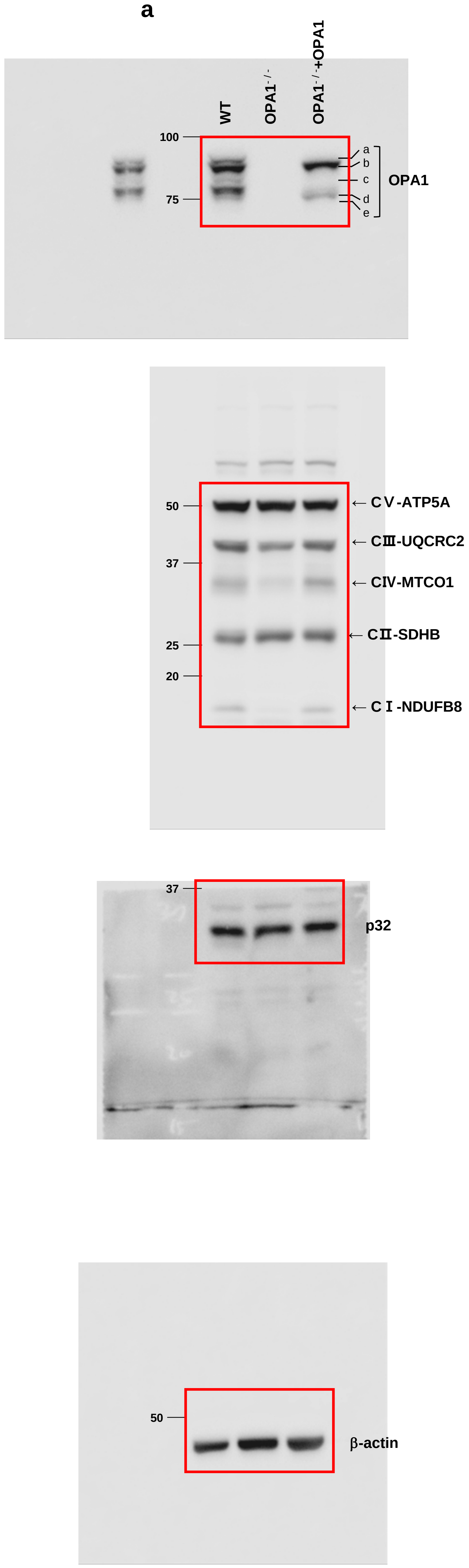

Supplementary Figure 7b

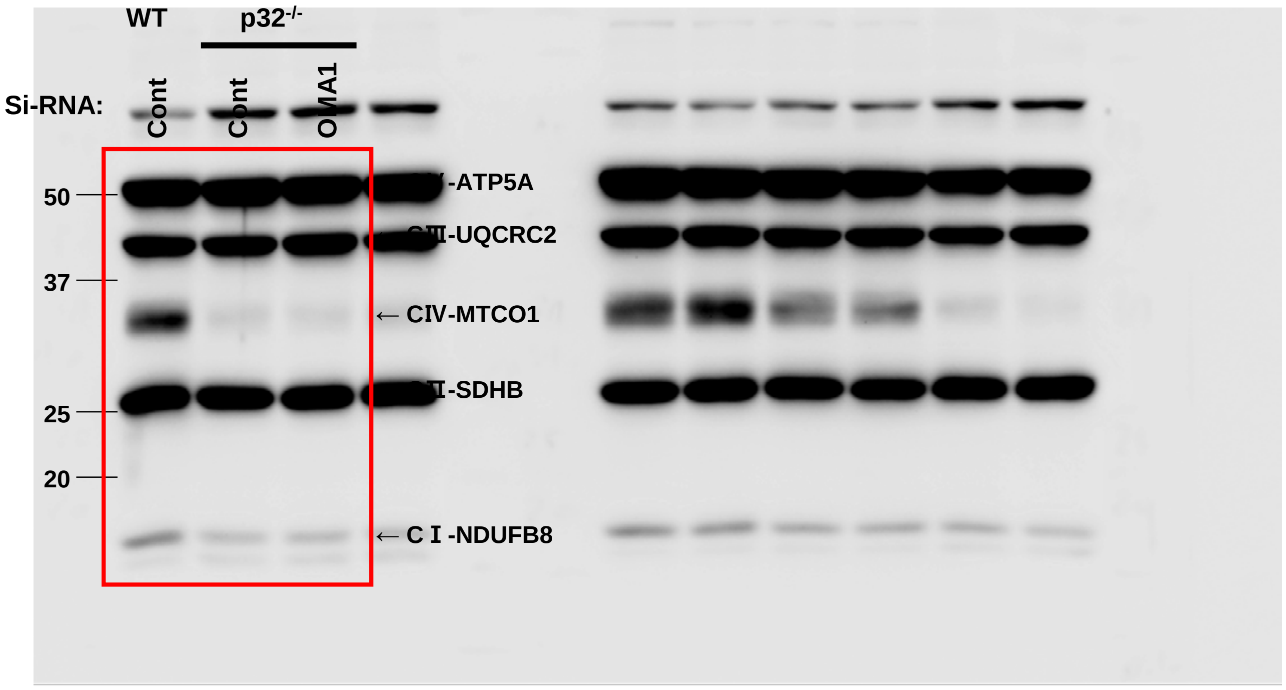

Supplementary Figure 7b

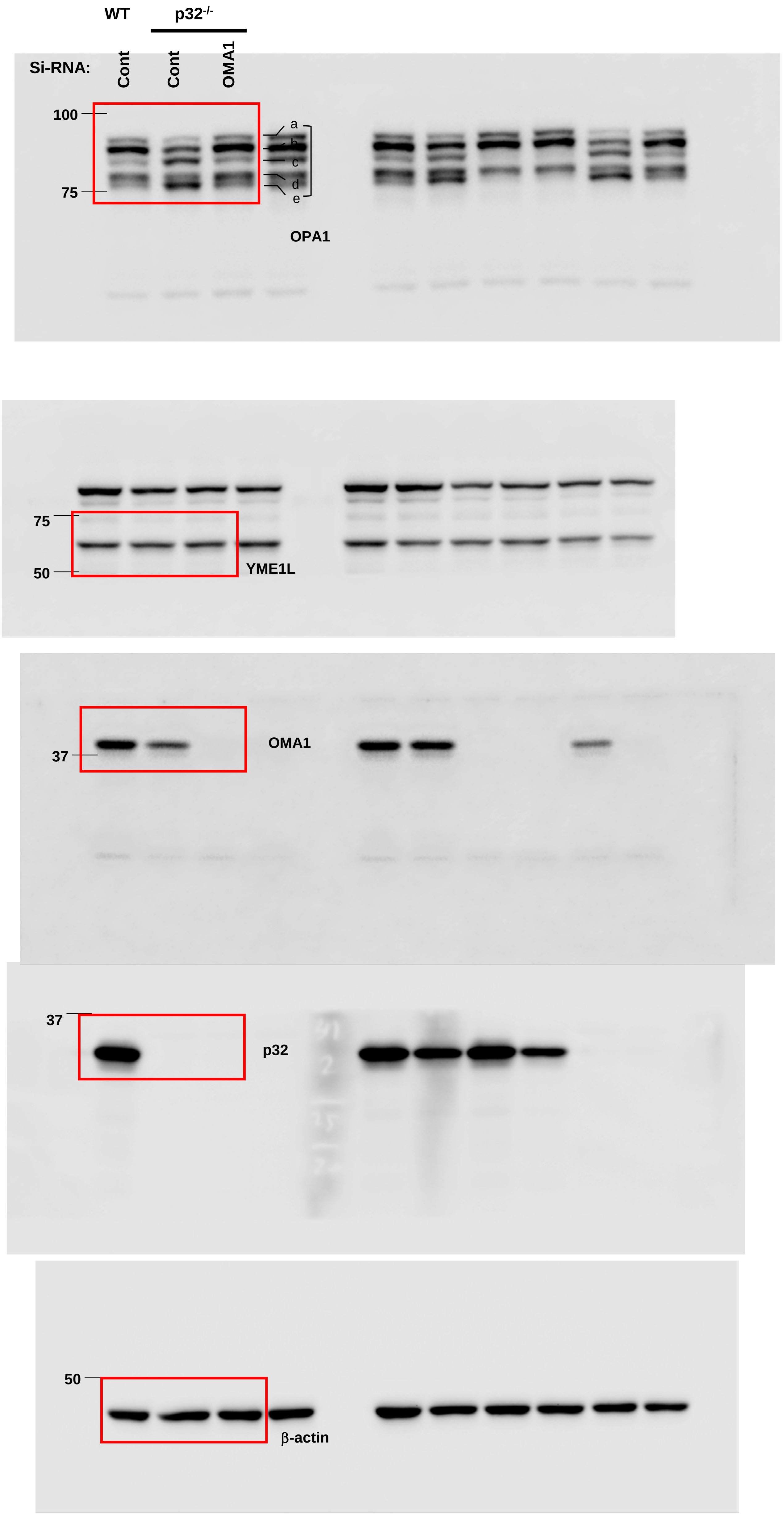

Supplementary Figure 12a

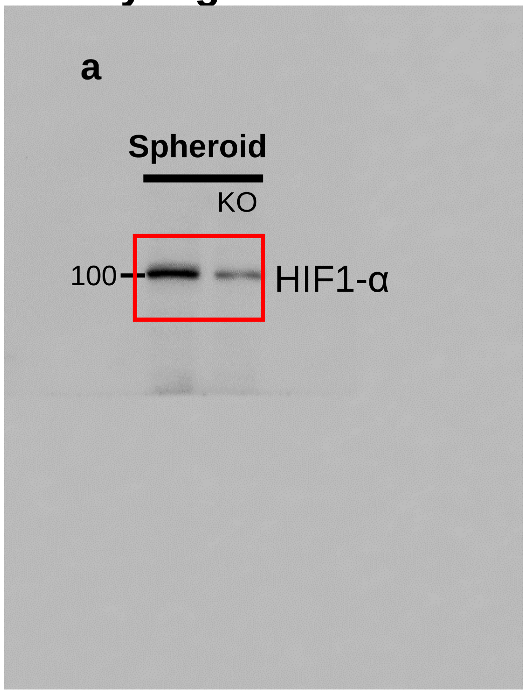

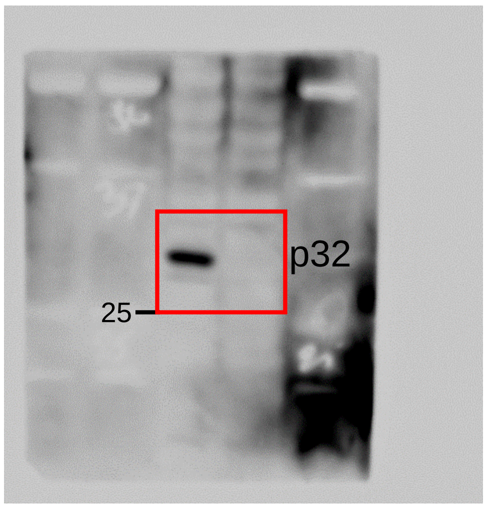

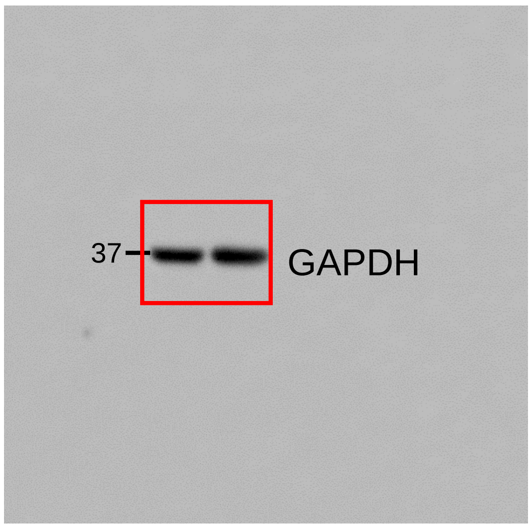

Supplementary Figure 12b

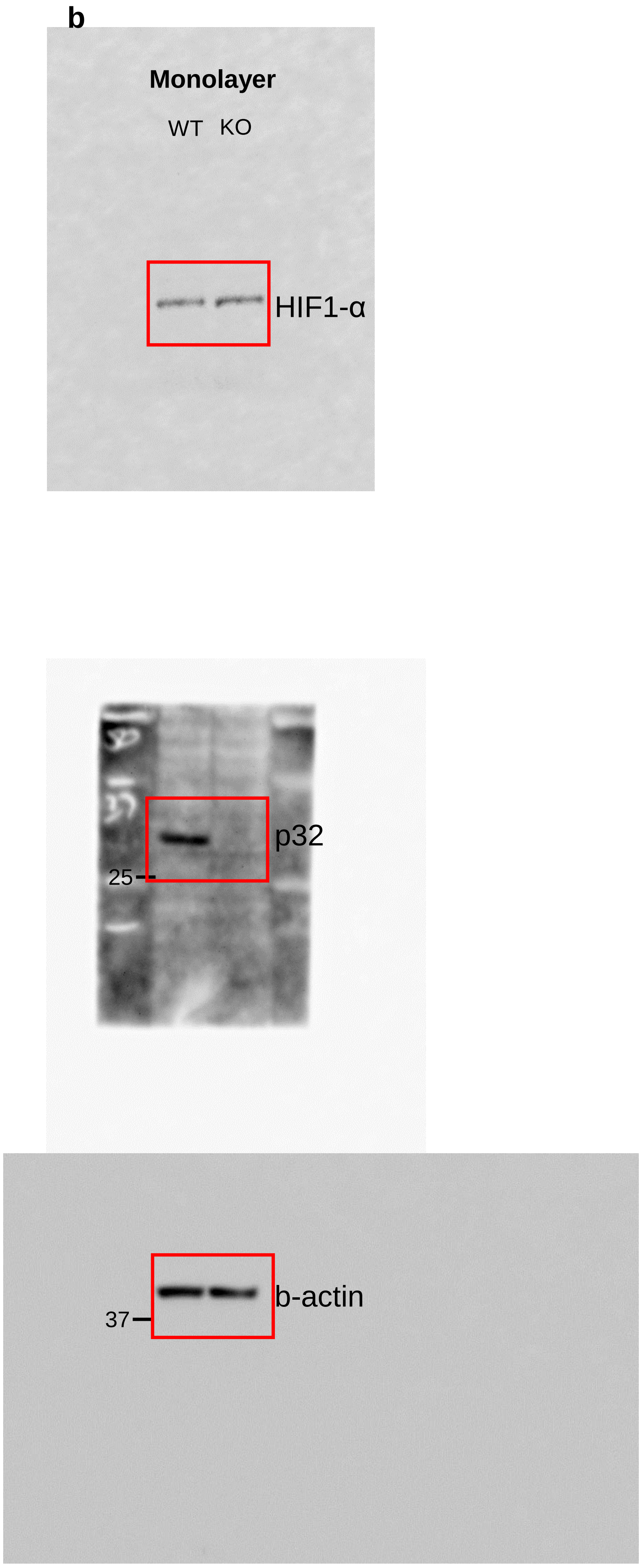

Supplementary Figure 13

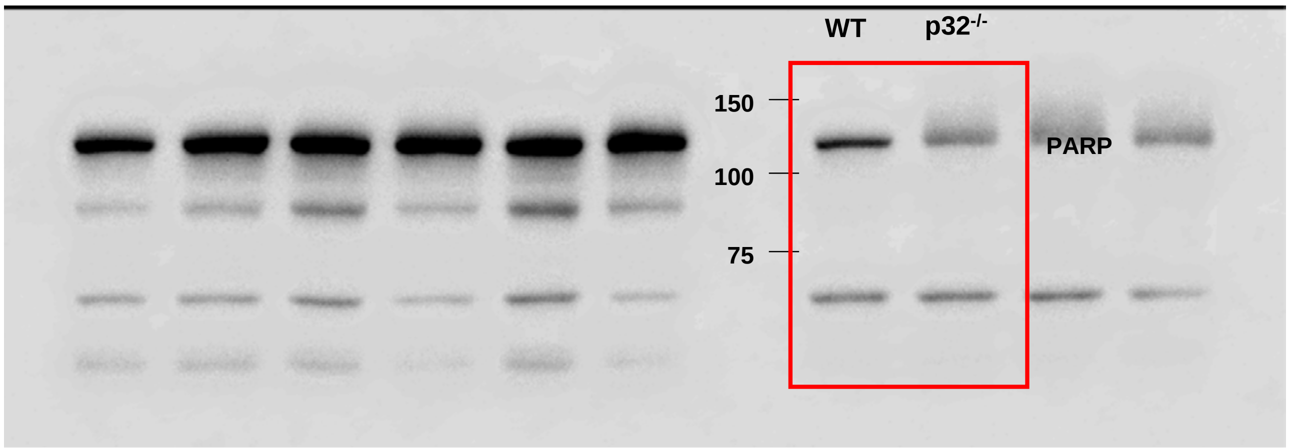

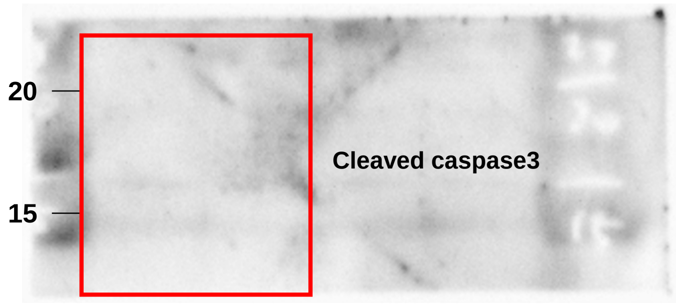

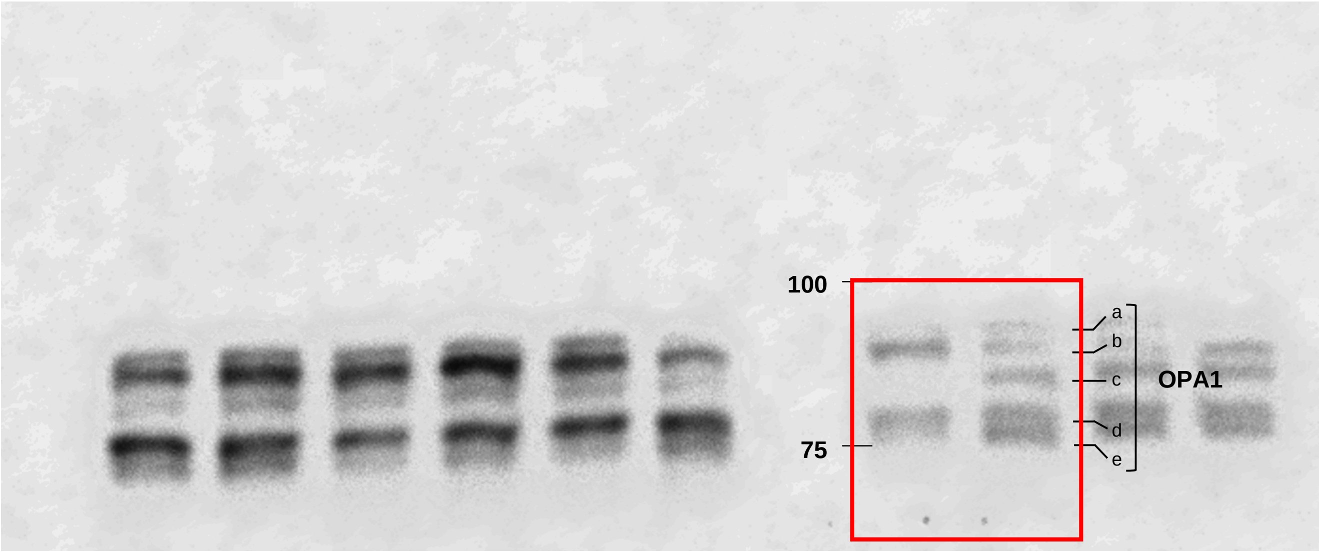

Supplementary Figure 13

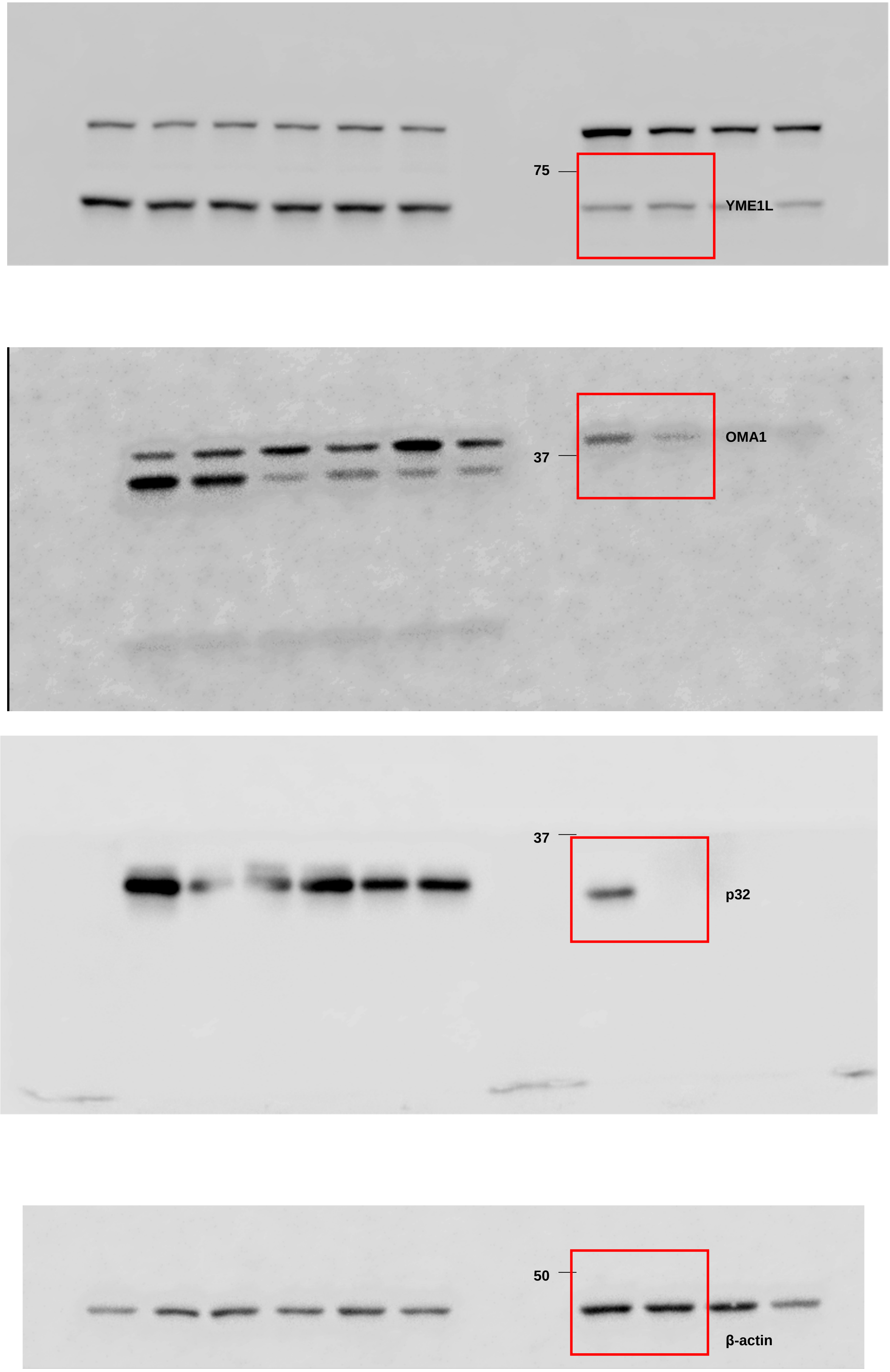

Supplement: Supplementary file 1 — Supplementary file1 (PDF 6131 kb) [file 41598_2020_67457_MOESM1_ESM.pdf]
